# Pan-cancer gene expression analysis of tissue microarray using EdgeSeq oncology biomarker panel and a cross-comparison with HER2 and HER3 immunohistochemical analysis

**DOI:** 10.1371/journal.pone.0274140

**Published:** 2022-09-22

**Authors:** Koichiro Inaki, Tomoko Shibutani, Naoyuki Maeda, Serenella Eppenberger-Castori, Stefan Nicolet, Yuki Kaneda, Kumiko Koyama, Yang Qiu, Kenichi Wakita, Masato Murakami

**Affiliations:** 1 Translational Science Department I, Daiichi Sankyo Co., Ltd., Tokyo, Japan; 2 Translational Research Department, Daiichi Sankyo RD Novare Co., Ltd., Tokyo, Japan; 3 Pathology, Universitätsspital Basel, Basel, Switzerland; 4 Global Oncology R&D, Daiichi Sankyo, Inc., Basking Ridge, NJ, United States of America; King Faisal Specialist Hospital and Research Center, SAUDI ARABIA

## Abstract

Molecular and protein biomarker profiling are key to oncology drug development. Antibody-drug conjugates (ADCs) directly deliver chemotherapeutic agents into tumor cells based on unique cancer cell biomarkers. A pan-cancer tissue microarray (TMA) data set and gene panel were validated and gene signature analyses were conducted on normal and cancer tissues to refine selection of ADC targets. Correlation of mRNA and protein levels, and human epidermal growth factor receptor (HER) expression patterns were assessed. An EdgeSeq biomarker panel (2862 genes) was used across 8531 samples (23 solid cancer types/subtypes; 16 normal tissues) with an established TMA data set, and immune cell and cell cycle gene signatures were analyzed. Discriminating gene expression signatures were defined based on pathological classification of cancer subtypes. Correlative analyses of HER2 and HER3 mRNA (EdgeSeq) and protein expression (immunohistochemistry **[IHC]**) were performed and compared with publicly available data (The Cancer Genome Atlas **[TCGA]**; Cancer Cell Line Encyclopedia **[CCLE]**). Gene expression patterns among cancer types in the TMA (EdgeSeq) and TCGA (RNA-seq) were similar. EdgeSeq gene signature analyses aligned with the majority of pathological cancer types/subtypes and identified cancer-specific gene expression patterns. TMA IHC H-scores for HER3 varied across cancer types/subtypes. In a few cancer types, HER3 mRNA and protein expression did not align, including lower liver hepatocellular carcinoma IHC H-score, compared with mRNA. Although all TNBC and ovarian cancer subtypes expressed mRNA, some had lower protein expression. This was seen in TMA and TCGA data sets, but not in CCLE. The EdgeSeq TMA data set can expand upon current biomarker data by including cancers not currently in TCGA. The primary analysis of EdgeSeq and IHC comparison suggested a unique protein-level regulation of HER3 in some tumor subtypes and highlights the importance of investigating protein levels of ADC targets in both tumor and normal tissues.

## Introduction

Molecular profiling and biomarker assessment from patient tissue and blood samples can help guide therapy selection, better define a patient’s prognosis and more accurately predict responses to specific treatments [1]. In recent years, major therapeutic advances have been made for cancer treatment, including biomarker-driven and targeted therapies for lung, colorectal, gastric and several difficult-to-treat cancers [2]. Antibody drug conjugate (ADCs) technology is one of these advances that has greatly progressed, emerging as a promising directed therapy for solid tumors [3]. ADCs are agents composed of a monoclonal antibody linked to a chemotherapeutic agent designed to deliver the cytotoxic payload to tumor cells while minimizing off-target toxicities. Targets of ADCs are plasma membrane proteins, ideally with higher expression in cancer cells than normal tissues. Particular targets of interest for ADCs are human epidermal growth factor receptor (EGFR/HER) family of genes (e.g., *EGFR*, *HER2*, *HER3*) [4–7] with an alteration that is often associated with the initiation and maintenance of tumor growth [8].

Pan-cancer gene and protein expression profile data are valuable in drug development, including selection of ADC targets, overall drug target discovery and drug indication consideration. Publicly available reference data of gene expression profiles, such as RNA-seq data from The Cancer Genome Atlas (TCGA) Program [9], can be used to determine therapeutic genes-of-interest. Although TCGA includes over 30 cancer types, several rare cancer types are not included, and information of gene–protein expression associations is limited. There is a need for additional and alternative resources to confirm and expand on that included in TCGA.

Here, we describe data from a pan-cancer reference data set of gene expression levels from 2376 tissue microarray (TMA) samples, including solid cancer types/subtypes and normal tissues, using a custom panel (2862 genes) of HTG EdgeSeq Oncology Biomarker Panel (OBP) platform, in which genes-of-interest were added to the original panel. The OBP has the sensitivity and dynamic range of next-generation sequencing and includes 24 gene groups/pathways and 17 key drug targets (i.e., CTLA-4, HER2, HER3, MET, PD-1, PD-L1) [10]. The EdgeSeq system has been used to identify cancer–related gene signatures in samples of lung [11], breast [12], colorectal [13], and bladder cancers [14].

The first objective of this analysis was to validate the TMA EdgeSeq platform against RNA-seq. Once validated, this platform was used to assess gene signature patterns based on pathological cancer types/subtypes present within the TMA data set, including several cancers not included in TCGA. Gene signatures related to immune cells, the cell cycle and other oncology-related genes were analyzed across cancers within TMA. Comparison of mRNA and protein expression of HER3 was also conducted to better understand the gene/protein regulation of this therapeutic target. Overall, these TMA reference data are a new and extensive resource for genes of interest for ADCs and targeted therapies.

## Materials and methods

### Data collection/ethical considerations

Clinical and histopathology data were collected retrospectively in a non-stratified manner. Data included patient age, primary cancer site, TNM stage, disease-specific survival, histological subtype, presence of vascular invasion and tumor diameter and grade. Approval for tissue collection, use of samples and data for the construction of this data set was obtained from the locally responsible ethics committees (Ethikkommission Norwest- und Zentralschweiz [EKNZ] Permission 2017–00302). The majority of the patients donating surgical tissues for the construction of the TMAs provided written informed consent approving research studies. For the one patient without a written informed consent, the ethics committee gave permission for tissue use. Tissue samples were coded and all related clinical data were provided in an anonymous manner to HTG molecular Diagnostic and Daiichi Sankyo.

### Tissue microarray

A pan-cancer TMA data set was established from specimens at the Biobank at the Institute of Pathology, University Hospital Basel, Switzerland. Thirty-one TMA blocks of non-consecutive primary cancer specimens and ~30% paired non-malignant adjacent tissue specimens were constructed by using TMA-Grand Master^®^ (3DHisteck; Sysmex AG, Switzerland). For TMA construction, formalin-fixed, paraffin-embedded tissue blocks were prepared according to standard protocols [15]. A total of 3017, 1-mm core specimens were processed. The percentage of tumor cells within each core sample was >50% for all tumor specimens. The commercially available Molar Chemicals KFT (Halàsztelek, àrpàd utca 1, H2314 Hungary) with quality certificate and melting point at 53°C–62°C was used. Overall, the 31 TMAs consisted of 2988 cores collected from 2492 patients. Two TMAs were dedicated to non-malignant tissues, the remaining 29 TMAs were subdivided according to the cancer type/subtype. Lung cancer subtypes were defined as described in Supporting Information.

### Gene expression analysis (EdgeSeq) of TMA

From each TMA core, four slides (4 μm) and one hematoxylin and eosin (H&E) slide were sent to HTG Molecular Diagnostics (Tucson, AZ, USA) for gene expression analysis termed as EdgeSeq, based on probe-based RNA counting by next generation sequencing (https://www.htgmolecular.com/assays/obp). A custom panel of 2867 probes, including 2560 HTG’s OBP genes, was designed. The next generation sequencing data were processed at HTG and included count data for all gene-specific probes and internal controls (Supporting Information).

### EdgeSeq data processing

Quality filters were applied to exclude the following samples with <1.5 million total read count or <0.1 of relative standard deviation of all probes (Supporting Information). Background signal from negative controls (e.g., insufficient digestion of non-hybridized probes) was subtracted from all gene probes as well as undergoing upper-quartile (UQ) normalization to reduce tissue-specific bias of the expression data (Supporting Information). Log transformed values (log_2_ [adjCPM-UQ + 1]) were calculated and used for the analyses. An empirical parameter was set to reduce data of low-quality samples from the final data set using house-keeping gene expression levels. Correlations (*R*) between each sample data and an average of universal RNA (uRNA) gene expression levels were calculated from 89 samples that passed the sequencing filter. Samples with low correlations (*R* < 0.4) were considered low quality and a sample quality filter was applied by empirically setting an exclusion parameter based on average of expression values of nine housekeeping genes (Supporting Information). Through these processes, we obtained 2479 data samples (2376 TMA, 88 uRNA controls and 15 multiple tissue controls) for 2867 probes. For the described analyses, five probes/genes among 2867 were not used for comparison of the EdgeSeq data with the Human Genome build 38 (hg38) data set used by TCGA and in-house RNA-seq (Supporting Information).

### TCGA RNA-seq data and gene signature analysis

RNA-seq data for TCGA were downloaded from UCSC-Xena (https://xenabrowser.net/). Some probes were not used in the comparison of EdgeSeq with TCGA or with in-house RNA-seq data because of circumstances described in Supporting Information. Comparisons were based on gene symbol with Ensemble Gene-ID. Gene annotations for cell type gene signature analysis were provided by HTG and public data (i.e., cancer-associated fibroblast [CAF] data from https://www.ncbi.nlm.nih.gov/pubmed/29198524) (S1 and S2 Files). Z-scores were calculated for all samples of a given gene and an average of these was considered as the gene signature score for each sample set. For cancer subtype gene signature analyses, subtype-discriminating genes were defined based on pathological subtype annotation (S3–S7 Files) and subtype scores, per sample (subtype gene average of Z-scores among all TMA samples). Each gene expression subtype, per sample, was defined by taking a maximum of subtype scores.

### Cell line culture and RNA analysis

Cancer cell lines (S1 File) were harvested at log phase growing points. RNA purification was performed with the AllPrep DNA/RNA Mini Kit (QIAGEN, Hilden, Germany). RNA quality was determined based on RNA integrity number, measured by Bioanalyzer or TapeStation (Agilent Technologies, Santa Clara, CA, USA). The same aliquot of RNA was used for RNA-seq analysis or was sent to HTG for EdgeSeq analysis.

For RNA-seq analysis, mRNA was purified from 1 μg of total RNA using NEBNext Poly(A) mRNA Magnetic Isolation Module (New England Biolabs, Ipswich, MA, USA). Complementary DNA (cDNA) was generated using NEBNext Ultra RNA Library Prep Kit for Illumina (New England Biolabs) (Supporting Information). EdgeSeq raw data (94 cell line samples) were processed in the same manner as TMA data (sequence quality filter, followed by adjustment of CPM, UQ-normalization and sample quality filter) and correlations, slopes and intercepts between RNA-seq data (log_2_ [TPM-UQ + 1]) and EdgeSeq data (log_2_ [adjCPM-UQ + 1]) were calculated.

## Results

### Correlation of EdgeSeq and RNA-seq gene expression data using cancer cell lines

RNA purified from 94 cancer cell lines was used to determine the degree of correlation between the EdgeSeq OBP, including probes for 2862 genes, and RNA-seq. The average data of these cell lines suggested an overall correlation (*R* > 0.4 and approaching 1.0) for most genes, including *HER2*, *HER3* and *PD-L1* (Supporting Information [S1A Fig in S8 File]). A biased expression pattern was observed with some genes/probes (see Supporting S1 File for probe sequences), such as *PPIA*, *PABPC1*, *RPL13*, *NPM1*, *HSP90AA1*, *DDX39B*, *XRCC6* and *BSG* displaying lower signals for EdgeSeq than RNA-seq (Supporting Information [S1B Fig in S8 File]). In contrast, other genes, such as *HIST1H3H*, *INS*, *GNAI3*, *PTTG2*, *HNF1A*, *H3F3C* and *CAMP*, demonstrated higher signals in EdgeSeq. Other oncogenic markers also demonstrated positive correlations between RNA-seq and EdgeSeq, with wide dynamic ranges (Supporting Information [S1C Fig in S8 File]). Correlations and dynamic ranges of some genes (e.g., immune-related genes) could not be accurately assessed because of low-expression levels in the cancer cell lines analyzed.

### Comparisons of TMA EdgeSeq and TCGA RNA-seq gene expression profiles

**Fig 1:**
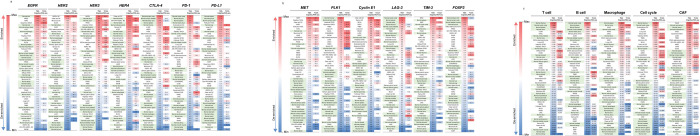
Gene expression patterns in tissue microarray (TMA; EdgeSeq) versus The Cancer Genome Atlas (TCGA; RNA-seq). Correlations in gene expression (log_2_ [adjCPM-UQ + 1]) by cancer type/subtype or in normal tissue for TMA EdgeSeq and TCGA RNA-seq were made for (**A**) human epidermal growth factor receptor (HER) family genes and immune-oncology markers of interest, (**B**) oncogenes of interest and (**C**) immune cell genes, cell cycle genes and cancer-associated fibroblast (CAF)-associated gene signatures. Expression is color-coded based on whether counts are approaching maximum (red) or minimum (blue) levels of detection. BC, breast cancer; EGFR, epidermal growth factor receptor; PD-L1, programmed death ligand-1. BLCA, bladder carcinoma; COADREAD, colorectal adenocarcinoma; ER, estrogen receptor; ESCA, esophageal cancer; HNSC, head-neck squamous cell carcinoma; KIRC, kidney renal clear cell carcinoma; KIRP, kidney renal papillary cell carcinoma; LCC, large cell carcinoma; LIHC, liver hepatocellular carcinoma; LUAD, lung adenocarcinoma; LUSC, lung squamous cell carcinoma; OC, ovarian cancer; PAAD, pancreatic adenocarcinoma; PD-1, programmed death protein 1; PD-L1, programmed death-ligand 1; PR, progesterone receptor; PRAD, prostate adenocarcinoma; THCA, thyroid cancer; TNBC, triple-negative breast cancer; UCEC, uterine corpus endometrial carcinoma.

Gene expression profiles of *HER* family genes, oncogenes and immune-oncology–related markers from TMA EdgeSeq were compared with those from TCGA. High expression of *HER2* was observed in both data sets for HER2+ breast cancer, urothelial bladder carcinoma and kidney renal papillary cell carcinoma. *HER3* was highly expressed in skin cutaneous melanoma, prostate adenocarcinoma, intestinal-stomach adenocarcinoma and estrogen receptor-positive (ER+), progesterone receptor-positive (PR+), HER2− breast cancer (ER+/PR+/HER2−), while generally lower in normal tissues (Fig 1A). *HER4* and *EGFR* demonstrated similar expression patterns among the two data sets. Oncogenes, such as *MET*, *PLK1* and *CCNE1*, demonstrated higher expression levels in cancer tissues than in normal tissues in the TMA and TCGA data sets.

Expression patterns for TMA EdgeSeq and TCGA RNA-seq were compared for genes related to the immune system, cell cycle and CAFs. Overall, there were similarities in expected patterns (Fig 1B and 1C). EdgeSeq data identified that normal hematopoetic tissues (i.e., spleen, thymus, lymph nodes) not included in TCGA had high expression of T cells, B cells and macrophage genes. Cancers with high tumor-infiltrating lymphocytes (e.g., lung cancers) had high expression of immune-oncology markers, such as *PD-L1*, *TIM3*, *FOXP3*, *PD-1*, *CTLA4*, and *LAG3* in TMA EdgeSeq and TCGA RNA-seq; this was also observed in normal hematopoietic tissues with TMA EdgeSeq (Fig 1B). Cell cycle gene signatures were highest in normal thymus and lymph node tissue, followed by various cancer tissues (e.g., ovarian and lung). The CAF gene signature was highest in pancreatic adenocarcinoma, cholangiocarcinoma, squamous esophageal carcinoma and lung squamous cell carcinoma; it was also high in normal reproductive tissues (e.g., myometrium, uterus and endometrium) (Fig 1C).

### EdgeSeq TMA marker gene signature analyses within TMA cancer types/subtypes

Clinical details were used to define cancer subtypes for breast cancer, esophageal cancer, stomach cancer and thyroid cancer (Table 1 and Supporting Information). Marker genes were selected to discriminate among cancer subtypes based on pathology. Gene signature analyses were conducted to characterize molecular cancer subtypes defined by EdgeSeq gene expression and to further refine the subtype. Several of the cancer types assessed were unique to the EdgeSeq TMA platform and are not included in the current TCGA data set (i.e., large-cell lung carcinoma [LCC], including large cell neuroendocrine carcinoma [LCNEC], salivary cancer and the clear cell, endometrioid and mucinous ovarian cancer subtypes) (Table 1). Genes identified by TMA EdgeSeq to be differentially expressed by cancer type and subtype are listed in S2 File.

**Table 1:** Samples for TMA EdgeSeq, TCGA RNA-seq and CCLE RNA-seq comparison.

	TMA	TCGA	CCLE
	EdgeSeq	RNA-seq	RNA-seq
**Cancer type–subtype**			
Bladder carcinoma	96	411	24
Breast cancer, HER2+	80	170	15
Breast cancer, ER+/PR+/HER2–	143	489	12
Breast cancer, TNBC	62	123	20
Cholangiocarcinoma	37	36	8
Colorectal adenocarcinoma	169	638	55
Endometrial carcinoma–uterine corpus	76	548	28
Esophageal carcinoma–adenocarcinoma	30	80	2
Esophageal carcinoma–squamous	43	82	24
Head-neck squamous cell carcinoma	69	502	30
Kidney–renal clear cell carcinoma	40	535	19
Kidney–renal papillary cell carcinoma	45	289	2
Liver hepatocellular carcinoma	104	374	23
Lung–adenocarcinoma	74	526	67
Lung–squamous cell carcinoma	73	501	21
Lung–large cell carcinoma	38		17
Skin cutaneous melanoma	33	471	49
Ovarian carcinoma–clear cell	8		9
Ovarian carcinoma–endometrioid	34		4
Ovarian carcinoma–mucinous	14		6
Ovarian carcinoma–serous	110	379	23
Pancreatic adenocarcinoma	61	178	40
Prostate adenocarcinoma	128	499	7
Salivary gland cancer	75		2
Sarcoma	66	263	30
Stomach adenocarcinoma–diffuse	40	73	19
Stomach adenocarcinoma–intestinal	64	167	18
Thyroid cancer–follicular	13		4
Thyroid cancer–papillary	23	504	2
**Normal tissue**			
Biliary duct	5	9	
Breast	43	113	
Colon	58	51	
Endometrium	15	35	
Esophagus	40	11	
Heart	3		
Ileum	9		
Kidney	26	104	
Liver	56	50	
Lung	63	108	
Lymph node	11		
Myometrium	8		
Pancreas	26	4	
Prostate	35	52	
Salivary Gland	10	44	
Skeletal muscle	9		
Skin	7	1	
Smooth muscle	10	2	
Spleen	8		
Stomach	34	32	
Testis	8		
Thymus	9		
Thyroid	20	58	
Urinary bladder	9	19	
Uterus	6		
Total	2376	8531	580

CCLE, Cancer Cell Line Encyclopedia; ER, estrogen receptor; HER, human epidermal growth factor receptor; HR, hormone receptor; PR, progesterone receptor; TCGA, The Cancer Genome Atlas; TMA, tissue microarray; TNBC, triple-negative breast cancer.

The TMA includes four subtypes of ovarian cancer: clear cell (*n* = 8), endometrioid (*n* = 34), mucinous (*n* = 14) and serous (*n* = 110); the first three are not a component of TCGA. Genes selectively over- or under-expressed in each subtype among the ovarian cancer samples were assessed (S3 File). The signature genes (highly expressing subtypes) included known markers, such as *CEACAM6* (mucinous), *DKK1* (endometrioid), *HNF1B* (clear cell and mucinous), *PGR* (endometrioid), *TFF3* (mucinous) and *WT1* (serous) [16–18]. Alignment of the subtypes based on pathological versus gene expression profile was observed for clear cell, mucinous and serous ovarian cancer. Approximately half of the pathological endometrioid subtypes were defined as one of the other three ovarian cancer subtypes by gene signature (S3 File).

Lung cancer subtypes assessed for gene signature analysis were lung adenocarcinoma (LUAD; *n* = 74), lung squamous carcinoma (LUSC; *n* = 73), LCNEC (*n* = 15) and large-cell lung carcinoma not otherwise specified (LC-NOS; *n* = 23); normal lung tissue (*n* = 63) was also included. Marker genes were selected to differentiate LUSC versus LUAD (*p* < 0.005) and LCNEC versus LUSC and LUAD combined (*p* < 0.05). One goal of this analysis was to annotate LC-NOS samples into specific subtypes by gene signature. LC-NOS samples were redefined into either LUAD (*n* = 8), LUSC (*n* = 5), or LCNEC (*n* = 10) (S4 File). However, signals for these groups by gene signature were weaker than that observed based on pathological subtyping. Varied gene expression profiles were also observed in a principal component analysis of the LC-NOS samples (Supporting Information [S2A Fig in S8 File] and S4 File). Normal lung tissue displayed a gene expression profile most similar to the LUAD subtype.

The TMA data set includes two subtypes in thyroid cancer, papillary (*n* = 23) and follicular (*n* = 13), as well as normal thyroid tissue (*n* = 20); the majority of thyroid cancers are classified as papillary in TCGA. Marker genes for each subtype were selected based on higher and lower expression over the other subtype and normal thyroid tissues (S5 File). There were two follicular subtype-high expressing genes, *ESM1* (endothelial cell specific molecule 1) and *THBS4* (thrombospondin 4). Fifty-four genes were highly expressed in the papillary subtype, including *HER3*. Gene expression profiles and pathological cancer subtypes aligned. Two-thirds of normal thyroid tissue samples demonstrated a weak follicular gene expression profile, while the others did not show similarities to either papillary or follicular profiles.

Gastric cancer was separated into two major subtypes, diffuse (*n* = 64) or intestinal (*n* = 40), for the TMA data set. Marker genes for each subtype were selected based on higher and lower expression over the other subtype and normal stomach tissues (S5 File). Diffuse-high genes (*n* = 16) included three epithelial mesenchymal transition markers (HALLMARK signature: *DCN*, *FLNA* and *IGFBP4*), while 36 intestinal-high genes included many cell cycle and cytokine genes, such as *AURKA*, *CDC25A* and *CXCL1*. The gene signature analysis suggests overall concordance between gene signature profiles and pathological cancer subtypes, while normal gastric tissues displayed weak diffuse or intestinal gene expression profiles.

TNBC has previously been divided into subtypes (basal-like 1 and 2 [BL1 and BL2], luminal androgen receptor [LAR] and mesenchymal [M]) based on gene expression profiles; the analysis was applied to TNBC of TCGA [20]. TCGA data were used to select marker genes to discriminate the four TNBC subtypes from EdgeSeq panel genes (S6 File). Selected gene signature analysis was applied to TMA data and defined BL1 in 17 samples (27%), BL2 in 19 (31%), LAR in 12 (19%) and M in 14 (23%). Similar proportions were observed with TCGA (29%, 27%, 19% and 25% among 113 samples, respectively) using the gene signature.

TMA has a unique rare cancer data set of salivary gland cancer, which is not included in TCGA, including several malignant tumor subtypes (*n* = 75; acinic cell carcinoma [Ac], adenoid cystic carcinoma [Ad], basal cell adenoma [Ba] and mucoepidermoid carcinoma [Mu]), benign epithelial tumor subtypes (pleomorphic adenoma [Pl] and warthin tumor [Wa]) and normal salivary gland tissue (*n* = 10). Marker genes for each subtype were selected based on higher and lower expression over other subtypes and normal salivary gland tissues. Analysis suggested overall correspondence between pathological and gene signature subtypes for salivary gland cancer (S7 File). Similar gene expression profiles were observed for Ba and Pl subtypes, and several Pl samples were annotated as Ba by the gene signature analysis. Several genes were identified with high or low expression among salivary cancer subtypes. Genes identified as highly expressed in Ba and Pl included *FGF2*, *FGFR1*, *HIF3A*, *SFRP1*, *TGFBR3* and *WIF1*, whereas expression of *FGF2*, *FGFR1*, *HIF3A* and *WIF1* were low in the Ac subtype.

The Ad, Ba and Pl subtypes displayed low expression of immune markers such as *CD27* and *CD3G*, which was the converse for the Wa subtype (S7 File). The gene expression pattern in the Mu subtype differed from the aforementioned subtypes and had high expression of integrin/extracellular matrix genes (i.e., *COL1A1*, *FN1* and *ITGB6*) and cell cycle-related genes (i.e., *KIF2C* and *TOP2A*). These patterns (complementary profiles in Ad and Bl and a unique Mu profile) were reflected in principal component analysis (Supporting Information [S2B Fig in S8 File] and S7 File).

### Gene expression analysis for select genes/gene signatures in breast and salivary gland cancer

**Fig 2:**
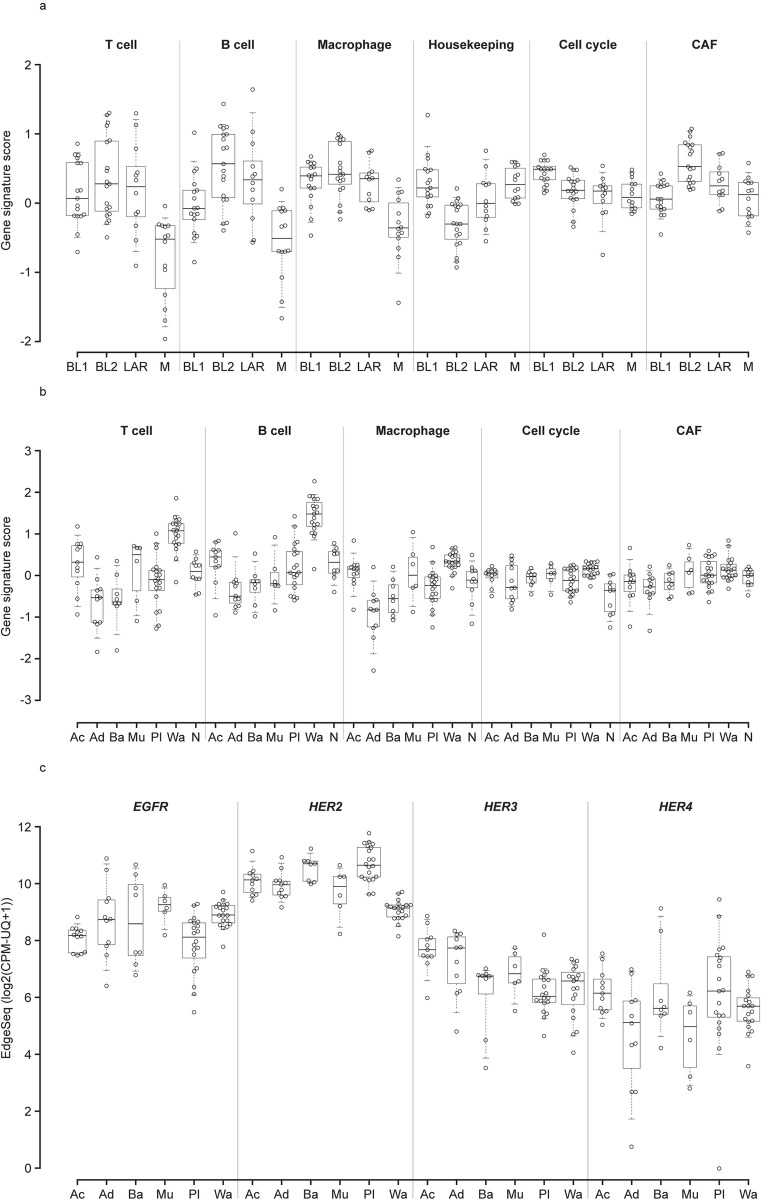
Gene signature analyses of breast and salivary gland cancer subtypes for genes and pathways for immune cells, the cell cycle and cancer-associated fibroblasts. Gene signature scores were determined for (**A**) TNBC and (**B**) salivary gland cancer subtypes for T cells, B cells, macrophages, cell cycle genes and CAF-related genes. (**C**) Expression of HER family genes was also determined for salivary gland cancer subtypes. For the box-and-whisker plot, the line in the box represents the median and whiskers indicate 5th and 95th percentiles. Ac, acinic cell; Ad, adenoid cystic; Ba, basal cell adenoma; BL, basal-like; EGFR, epidermal growth factor receptor; LAR, luminal androgen receptor; M, mesenchymal; Mu, mucoepidermoid; Pl, pleomorphic; Wa, Warthin tumor.

Gene signature scores for immune cells (T cells, B cells and macrophages), housekeeping genes, cell cycle genes and CAFs were determined for TNBC and salivary gland cancer subtypes (S2 File). For TNBC, immune cells had the lowest gene signature in the M subtype, which is in accordance with a previous report (Fig 2A and Supporting Information [S1 Table in S8 File]) [19]. The housekeeping gene signature was highest in the BL1 and M subtypes, and the CAF signature was notably higher in the BL2 subtype. The cell cycle gene signature was much higher in BL1, which has been reported elsewhere [19]. In salivary gland cancer subtypes, immune cell gene signatures were high (>0.6) in the Wa subtype, but lower (<0.0) in Ad, Ba and Pl subtypes (Fig 2B and Supporting Information [S2 Table in S8 File]). The cell cycle signature was generally higher in tumors than normal tissue.

Investigation of *HER* gene family member expression in salivary gland cancer showed that Mu and Wa subtypes had higher *EGFR* expression among the salivary gland cancer subtypes (Fig 2C). *HER2* was higher in Ba and Pl subtypes and lowest in Wa; *HER3* was higher in the Ac and Ad subtypes, and *HER4* was higher in the Ac and Pl subtypes. Additional assessment of specific immune-oncology marker expression revealed high expression levels for the Wa subtype and that the Ad subtype generally had the lowest expression of *PD-L1*, *TIM3*, *FOXP3* and *CTLA-4* (Supporting Information [S3 Fig in S8 File]).

### Correlative analyses of HER2 and HER3 mRNA and protein expression

**Fig 3:**
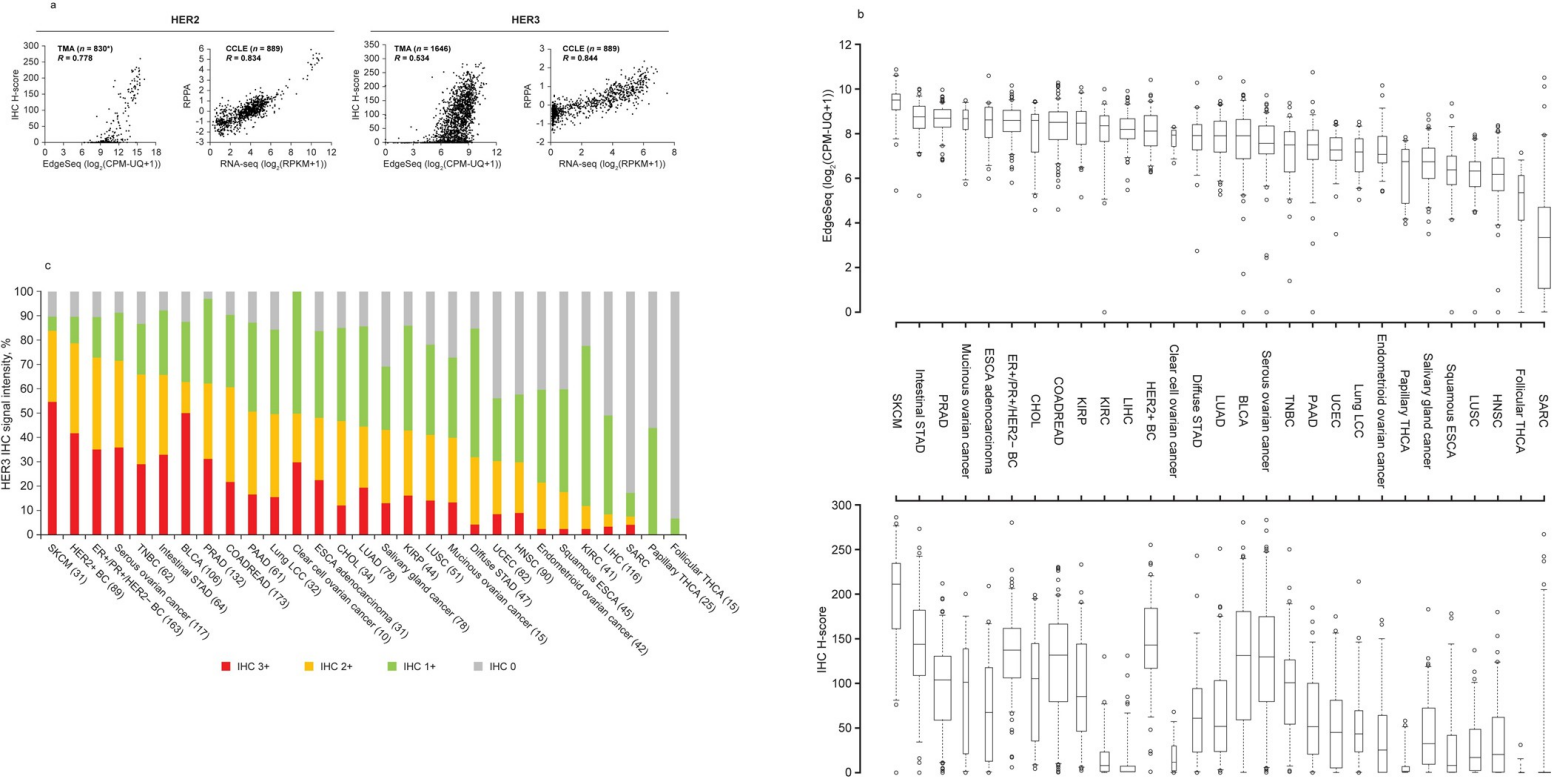
HER2 and HER3 mRNA and protein expression. (**A**) Correlation of mRNA and protein expression of HER2 and HER3 in TMA (via EdgeSeq and immunohistochemistry [IHC] H-score) and CCLE (via RNA-seq and RPPA) data sets. Correlation coefficients were calculated with *R* > 0 and approaching one signifying a positive correlation. No data were available for HER2-cancers using visual examination. (**B**) HER3 mRNA (EdgeSeq) and protein (IHC H-score) expression from TMA data set. (**C**) TMA cancer types/subtypes sorted by HER3 IHC positivity (IHC 3+ and 2+). IHC scores of 3+ and 2+ were considered signal intensity that was positive for HER3 expression. For the box-and-whisker plot, the line in the box represents the median and whiskers indicate 5^th^ and 95^th^ percentiles. BC, breast cancer.

Gene and protein expression patterns and correlations to the ADC targets, HER2 and HER3, were investigated within TMA and CCLE data sets for all cancer samples. mRNA and protein expression levels of HER2 and HER3, respectively, correlated in TMA (*R* = 0.778 and *R* = 0.534) and CCLE (*R* = 0.834 and *R* = 0.844) (Fig 3A).

Comparison of HER2 mRNA (EdgeSeq) and protein levels by H-score (Supporting Information [S4A Fig in S8 File] and Supporting Information) in the TMA data set defined simple correlations whereas differences in median HER3 EdgeSeq mRNA and protein (IHC H-score) levels varied by cancer type/subtype (Fig 3B). Cancers that demonstrated decreased HER3 protein versus mRNA expression included kidney renal clear cell carcinoma, liver hepatocellular carcinoma and clear cell ovarian cancer, whereas those with increased HER3 protein expression included HER2+ breast cancer, bladder carcinoma and serous ovarian cancer. Although liver hepatocellular carcinoma and clear cell ovarian cancer expressed *HER3* mRNA (Fig 3B), almost no HER3 protein expression was detected in these cancers in the TMA data set. Overall, prevalence of HER3 positivity by IHC score (IHC 2+ and 3+) among the tumor types/subtypes (Fig 3C) slightly differed from that by *HER3* EdgeSeq mRNA level (Fig 3B, top). Protein and mRNA correlations of HER3 were examined within the TMA and CCLE data sets for liver hepatocellular carcinoma and ovarian clear cell carcinoma. Although high mRNA and low protein expression of HER3 was detected for these cancer types in the TMA data set, this pattern was not observed in the CCLE data set of cell lines (Supporting Information [S4B Fig in S8 File]).

**Fig 4:**
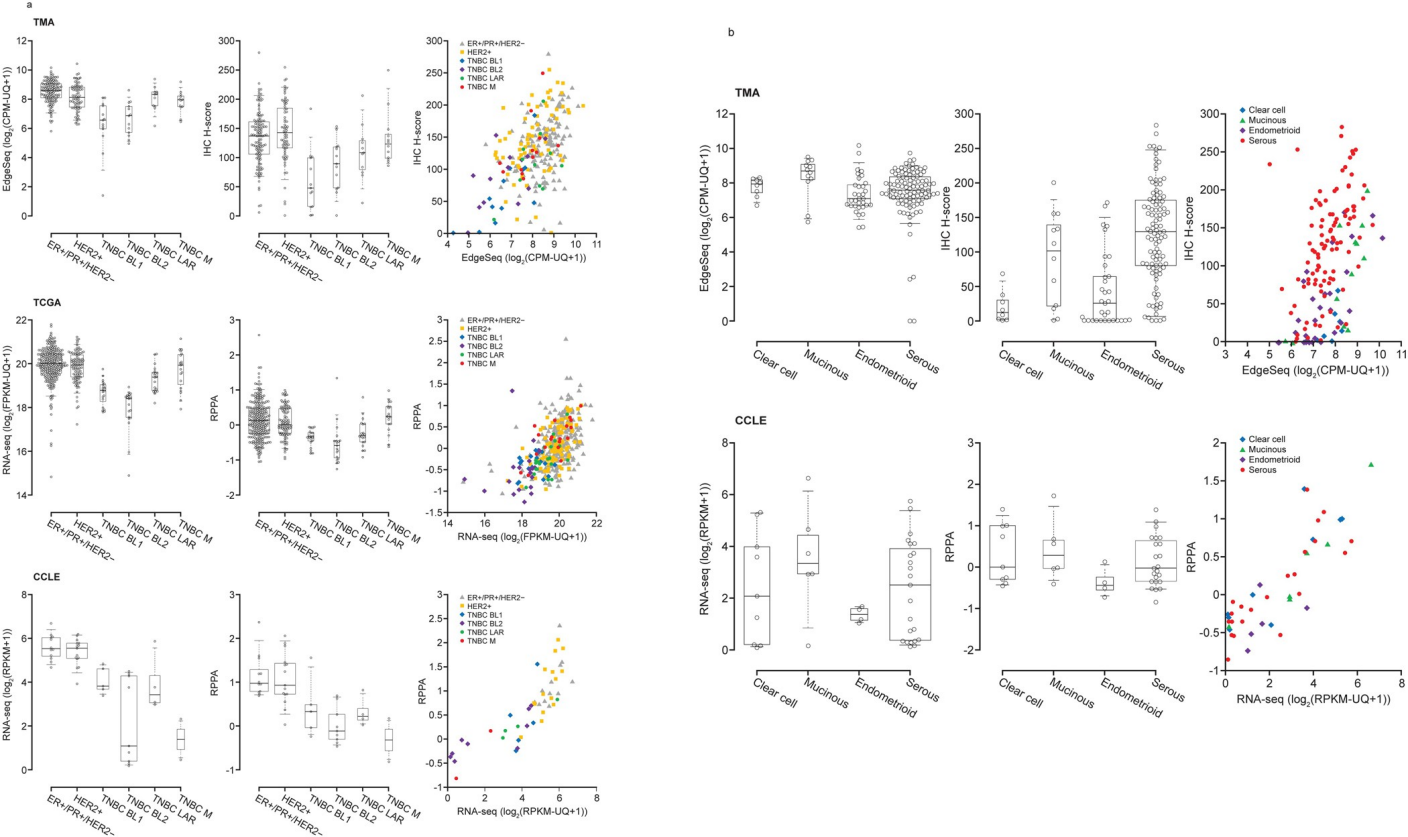
HER3 mRNA and protein expression levels in breast and ovarian cancer. (**A**) HER3 expression in breast cancer in TMA (EdgeSeq and IHC H-score), TCGA (RNA-seq and RPPA) and CCLE (RNA-seq and RPPA) data sets and in (**B**) ovarian cancer TMA (EdgeSeq and IHC H-score) and CCLE (RNA-seq and RPPA) data sets. The rightmost graph in each panel displays the mRNA:protein correlation plot for each respective cancer type and data set. For the box-and-whisker plot, the line in the box represents the median and whiskers indicate 5th and 95th percentiles.

Additional assessments were conducted across the TMA, TCGA and CCLE data sets in breast and ovarian cancer types/subtypes, based on the noted differences in HER3 mRNA and protein expression between in vitro (CCLE) and in vivo (TMA) data, as well as those between mRNA and protein expression. In TMA and TCGA data sets, expression levels of HER3 mRNA/protein in TNBC were higher in LAR and M than BL1 and BL2, with similar levels as ER+/PR+/HER2− and HER2+ breast cancer subtypes. However, the same expression patterns in TNBC were not observed in the CCLE data set (Fig 4A and Supporting Information [S3 Table in S8 File]). In ovarian cancer, *HER3* mRNA levels were highest in the mucinous subtype; however, protein levels (IHC H-score) were highest in the serous subtype (Fig 4B). Correlation of EdgeSeq and IHC H-score data suggests higher IHC scores relative to EdgeSeq mRNA levels for serous ovarian cancer than other ovarian cancer subtypes. *HER3* mRNA levels among ovarian cancer subtypes demonstrated similar expression patterns among the TMA and CCLE data sets. However, protein levels of HER3 were comparable among the ovarian cancer subtypes in the CCLE data set (Fig 4B and Supporting Information [S4 Table in S8 File]).

### Analysis of HER2 and HER3 co-expression

**Fig 5:**
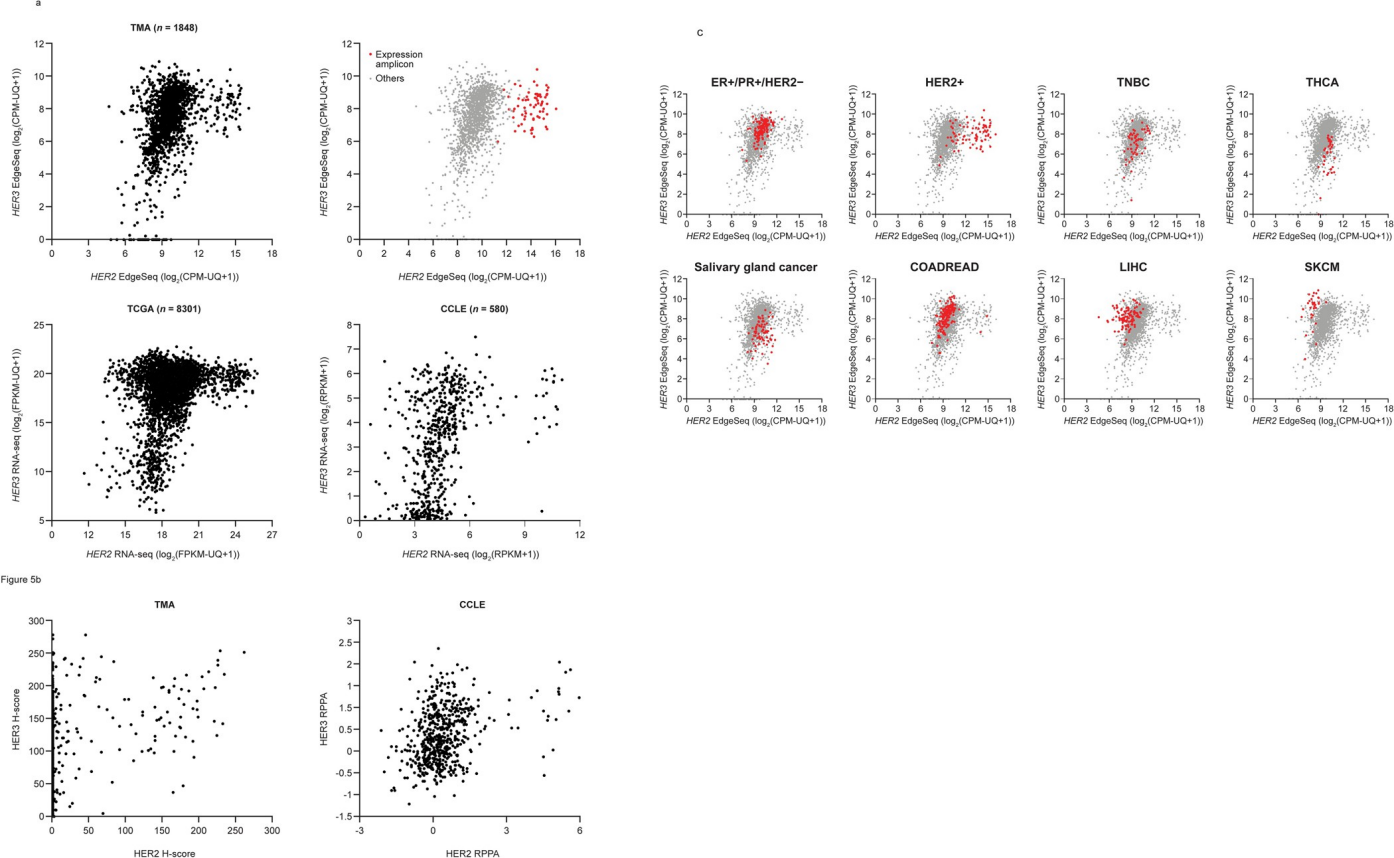
HER2 and HER3 co-expression. (**A**) *HER2* and *HER3* mRNA coexpression was assessed among all TMA EdgeSeq cancer samples (*n* = 1848) and matched samples for TCGA RNA-seq (*n* = 8301) and CCLE RNA-seq (*n* = 580); the *HER2* expression amplicon (red dots) had an average Z-score >1.5 and included the neighboring seven genes (+/- 51kb) of *HER2*; (**B**) HER2 and HER3 protein expression levels in TMA (IHC H-score) and CCLE (RPPA); (**C**) *HER2* and *HER3* mRNA in various tumor types from TMA EdgeSeq.

Correlation of *HER2* and *HER3* mRNA co-expression was analyzed among the TMA, TCGA and CCLE data sets (Fig 5A) to gain additional insight into the regulation of gene expression of these gene family members among the TMA cancer types/subtypes. Across data set comparisons, correlation was observed for *HER2* and *HER3* in lower ranges, while a portion of samples show extremely high *HER2* expression. In these *HER2* high samples, concurrent overexpression was observed in neighboring genes (termed the *HER2* expression amplicon) (Fig 5A top right, indicated in red). Correlation of co-overexpression of HER2 protein and neighboring genes within the *HER2* amplicon was also observed (*R* = 0.757; [S5 Fig in S8 File]). Analysis of HER2 and HER3 protein expression in CCLE RPPA data showed a similar correlation pattern of HER2 and HER3 to that of mRNA level. However, in TMA IHC data, high HER2 protein levels (H-score >100) were observed in a portion of samples (Fig 5B). Although most of them showed an amplified gene expression pattern in genes within the *HER2* expression amplicon, there was almost no correlation outside of the *HER2*-amplified range.

*HER2* and *HER3* mRNA co-expression also underwent correlation assessment in the various cancer types/subtypes. Three breast cancer subtypes displayed distinct patterns, which aligned with their known phenotypes: HER2+ breast cancer included HER2-genomic amplified samples; ER+/PR+/HER2− breast cancer included HER3 expression in a higher range; and HER2 expression was in the lower range in TNBC (Fig 5C). Correlation of EdgeSeq *HER2* and *HER3* mRNA expression patterns in additional tumor types/subtypes revealed high *HER2* expression in salivary gland tumors and thyroid carcinoma and high *HER3* expression in colorectal adenocarcinoma, liver hepatocellular carcinoma and skin cutaneous melanoma (Supporting Information [S6A Fig in S8 File]). Although these expression patterns by subtype were also observed for TCGA data, several CCLE cancer types showed different patterns from TMA and TCGA, such as thyroid carcinoma and liver hepatocellular carcinoma (Supporting Information [S6B Fig in S8 File]).

## Discussion

These data validate that the EdgeSeq TMA data set provides an additional resource for biomarker information across multiple cancer types/subtypes, including several that are not currently included in publicly available data sets, such as TCGA. In addition, gene signature analysis helped define expression of specific genes associated with certain pathologically defined cancers, potentially facilitating treatment decisions and prognosis formulation.

The characterization of cancer subtypes by gene signature described here will add molecular insight into the current classification of cancers by pathological subtype. Although most tumor types had alignment by known pathological type and EdgeSeq gene signature, there were some noted variations. Specifically, half of the tumor tissue defined as ovarian endometrioid cancer were epigenetically aligned with other ovarian cancer subtypes. The rationale for this outcome may be that fewer gene targets were used within the ovarian gene signature set and/or because of heterogenous gene expression within the specimens. For lung cancer, the identification of a LUAD gene expression profile in a majority of normal lung tissue may afford some background into the cell lineage and characteristics of this tumor subtype. In addition, TMA EdgeSeq data allowed for a more detailed classification of several LC-NOS samples into specific subtypes; however, these gene signature scores were low.

TMA EdgeSeq gene signature classification may also assist with improving targeted treatment selection and cancer prognosis predictions. For example, several genes related to fibroblast growth factor and WNT signaling pathway were highly expressed in specific salivary gland cancer subtypes, highlighting a potential therapeutic target. Furthermore, high CAF-gene signatures were identified in the BL2 TNBC subtype, which may be related to a worse prognosis in patients who are undergoing neoadjuvant chemotherapy.

Although the EdgeSeq-RNA-seq validation experiments demonstrated an overall correlation, some degree of saturation was observed. Biased expression patterns occurring with EdgeSeq may be related to the small probe size of 50 bp. A smaller probe may either result in lower signals because of lack of variant detection that can be detected by RNA-seq, and/or higher signals from nonspecific probe hybridization. In addition, some of the lower and higher biased expression may be due to EdgeSeq probe position among transcript variants and poly-A selection of the RNA-seq method, respectively.

HER2 and HER3 belong to the HER family of receptor tyrosine kinases and are commonly overexpressed in multiple tumor types [20–22]; their overexpression has been linked to poor clinical outcomes [23–25]. ADCs targeting HER2 and HER3, such as T-DXd and HER3-DXd, have demonstrated antitumor activity through in vitro and in vivo tumor models [6, 7, 26] as well as in patients with breast or lung cancer [27, 28]. As such, we investigated the gene and protein expression of HER2 and HER3 within the TMA data set as well as their degree of co-expression. Variations in expression of HER2 and HER3 among cancer types and subtypes supports development of specific directed therapies and will play a role in refining indications for approved agents. When comparing protein and mRNA expression of HER3 in cancer types across platforms, similar correlations were observed in several cancer types in TMA and TCGA, but not CCLE. As CCLE is an in vitro data set, these differences suggest in vivo and gene specific epigenetic regulation. The comparison of data in tissue and cell line data sets highlights the importance of examining protein levels as a component of ADC and other targeted drug development.

## Conclusions

Overall, the alignment of EdgeSeq and RNA-seq gene expression profiles for *HER* family genes, oncogenes and immune-oncology markers and the inclusion of additional cancer subtypes supports the use of the TMA EdgeSeq platform as a reference alternative and/or supplement to TCGA and CCLE in oncology research. The correlative analysis of HER2 and HER3 mRNA and protein expression, and the immune signature scores described here, will facilitate the development of ADCs directed to these proteins.

## Supporting information

S1 File TMA EdgeSeq probes, signature gene annotation, and cell line details. (XLSX)

S2 File TMA EdgeSeq and TCGA gene expression score averages by cancer type and gene signatures for immune cell, housekeeping, cell cycle, and cancer-associated fibroblast genes. (XLSX)

S3 File Ovarian cancer gene expression levels and gene signature profiles by pathological subtype. (XLSX)

S4 File Lung cancer gene expression levels, gene signature profiles by pathological subtype, and principal component analysis. (XLSX)

S5 File Thyroid and gastric cancer gene expression levels and gene signature profiles by pathological subtype. (XLSX)

S6 File Triple-negative breast cancer gene expression levels, gene signature profiles by pathological subtype, and HER2 amplicon expression profile by cancer type. (XLSX)

S7 File Salivary gland cancer gene expression levels, gene signature profiles by pathological subtype, and principal-component analysis. (XLSX)

S8 File (DOCX)
