# Supplementary material for: Pan-cancer gene expression analysis of tissue microarray using EdgeSeq oncology biomarker panel and a cross-comparison with HER2 and HER3 immunohistochemical analysis
Source: PLoS One. 2022 Sep 22;17(9):e0274140. doi: 10.1371/journal.pone.0274140 (PMC9498941; doi:10.1371/journal.pone.0274140)
Supplement: S8 File — (DOCX) [file pone.0274140.s008.docx]

**Supplementary Online Content**

Inaki K, Shibutani T, Maeda N, Eppenberger-Castori S, Nicolet S, Kaneda Y, Koyama K, Qiu Y, Wakita K and Murakami M. Pan-Cancer Gene Expression Analysis of Tissue Microarray Using EdgeSeq Oncology Biomarker Panel and a Cross-Comparison with HER2 and HER3 Immunohistochemical Analysis. *Cancers*.

**This supplementary material has been provided by the authors to give readers additional information about their work.**

The following Supplementary data spreadsheets are available online with the full text of the manuscript.

# Supplementary data 1. TMA EdgeSeq probes, signature gene annotation and cell line details

# Supplementary data 2. TMA EdgeSeq and TCGA gene expression score averages by cancer type, and gene signatures for immune cell, housekeeping, cell cycle and cancer-associated fibroblast genes

# Supplementary data 3. Ovarian cancer gene expression levels and gene signature profiles by pathological subtype

# Supplementary data 4. Lung cancer gene expression levels, gene signature profiles by pathological subtype and principal component analysis

# Supplementary data 5. Thyroid and gastric cancer gene expression levels and gene signature profiles by pathological subtype

# Supplementary data 6. Triple-negative breast cancer gene expression levels, gene signature profiles by pathological subtype and HER2 amplicon expression profile by cancer type

# Supplementary data 7. Salivary gland cancer gene expression levels, gene signature profiles by pathological subtype and principal-component analysis

**S1 Table.** EdgeSeq TMA immune cell gene signatures for TNBC subtypes.

|  | T cell | CD4 T cell | CD8 T cell | B cell | Macrophage | Housekeepers | AMPK pathway | Angiogenesis | Apoptosis | Cardiotoxicity | Cell cycle | Cluster of differentiation | DMPK | DNA repair | EGF/PDGF pathway | EGFR/HER pathway | FGFR pathway | Hedgehog pathway | Hypoxia | Immuno-oncology | JAK/STAT  pathway | MAPK pathway | NFκB pathway | PI3K/AKT  pathway | Stem cells | Stress toxicity | WNT pathway | CAF |
| --- | --- | --- | --- | --- | --- | --- | --- | --- | --- | --- | --- | --- | --- | --- | --- | --- | --- | --- | --- | --- | --- | --- | --- | --- | --- | --- | --- | --- |
| BL1 | 0.144 | 0.281 | 0.229 | -0.030 | 0.296 | 0.291 | -0.185 | -0.187 | -0.006 | -0.148 | 0.430 | -0.007 | -0.230 | 0.106 | -0.078 | -0.092 | -0.114 | -0.142 | -0.011 | 0.014 | -0.083 | 0.008 | 0.117 | -0.026 | -0.091 | 0.024 | -0.105 | 0.063 |
| BL2 | 0.363 | 0.476 | 0.486 | 0.536 | 0.472 | -0.312 | -0.008 | 0.304 | 0.187 | 0.347 | 0.177 | 0.336 | 0.085 | 0.082 | 0.057 | 0.109 | 0.140 | 0.291 | 0.177 | 0.358 | 0.153 | 0.106 | 0.235 | 0.040 | 0.224 | 0.154 | 0.224 | 0.595 |
| LAR | 0.205 | 0.226 | 0.262 | 0.338 | 0.280 | 0.050 | 0.027 | 0.043 | 0.048 | 0.085 | 0.088 | 0.112 | -0.079 | 0.081 | 0.073 | 0.026 | 0.009 | -0.088 | 0.046 | 0.050 | 0.048 | 0.026 | 0.031 | 0.055 | 0.011 | 0.013 | -0.020 | 0.281 |
| M | -0.808 | -0.429 | -0.563 | -0.560 | -0.339 | 0.281 | -0.275 | -0.278 | -0.264 | -0.132 | 0.121 | -0.392 | -0.430 | -0.107 | -0.093 | -0.181 | -0.203 | -0.305 | -0.030 | -0.447 | -0.316 | -0.082 | -0.376 | -0.100 | -0.311 | -0.215 | -0.199 | 0.065 |

Enriched (red) or de-enriched (blue) expression is color-coded for each gene signature by TNBC subtype.

AMPK, AMP-activated protein kinase; BL, basal-like; CAF, cancer-associated fibroblast; DMPK, dystrophia myotonica-protein kinase; EGFR, epidermal growth factor receptor; FGFR, fibroblast growth factor receptor; HER, human epidermal growth factor receptor; LAR, luminal androgen receptor;
M, mesenchymal; PDGF, platelet-derived growth factor; TNBC, triple-negative breast cancer.

**S2 Table.** EdgeSeq TMA immune cell gene signatures for salivary gland cancer subtypes.

|  | | T cell | CD4 T cell | CD8 T cell | B cell | Macrophage | Housekeeper | AMPK pathway | Angiogenesis | Apoptosis | Cardiotoxicity | Cell cycle | Cluster of differentiation | DMPK | DNA repair | EGF/PDGF pathway | EGFR/HER pathway | FGFR pathway | Hedgehog pathway | Hypoxia | Immuno-oncology | JAK/STAT pathway | MAPK pathway | NFκB pathway | PI3K AKT pathway | Stem cells | Stress toxicity | WNT pathway | CAF |
| --- | --- | --- | --- | --- | --- | --- | --- | --- | --- | --- | --- | --- | --- | --- | --- | --- | --- | --- | --- | --- | --- | --- | --- | --- | --- | --- | --- | --- | --- |
| Ac | 0.26883 | | 0.176145 | 0.125451 | 0.320494 | 0.076176 | 0.424091 | 0.282465 | -0.00787 | 0.179291 | -0.05093 | -0.02009 | 0.109824 | -0.0462 | 0.218501 | 0.218989 | 0.181752 | 0.214466 | 0.102921 | 0.0213 | 0.144936 | 0.14666 | 0.158696 | 0.147746 | 0.277213 | 0.062884 | 0.150114 | 0.075067 | -0.18591 |
| Ad | -0.65178 | | -0.81604 | -0.54469 | -0.33625 | -0.92274 | -0.26204 | -0.22445 | -0.26023 | -0.24451 | -0.43139 | -0.18092 | -0.47903 | -0.40115 | -0.04555 | -0.12419 | -0.11192 | -0.07968 | 0.000885 | -0.38262 | -0.41743 | -0.36676 | -0.17212 | -0.56889 | -0.23598 | -0.07156 | -0.29276 | -0.15329 | -0.32162 |
| Ba adenoma | -0.56785 | | -0.65014 | -0.45258 | -0.24305 | -0.51911 | 0.075738 | 0.05442 | -0.12314 | -0.0187 | -0.18684 | -0.07523 | -0.22062 | -0.191 | 0.125299 | 0.166524 | 0.165271 | 0.26539 | 0.384174 | -0.18986 | -0.15064 | -0.16219 | 0.10292 | -0.29585 | 0.18235 | 0.081269 | -0.07663 | 0.141481 | -0.16347 |
| Mu | 0.114387 | | 0.328031 | 0.211879 | -0.06475 | 0.064534 | 0.164429 | -0.11751 | 0.071068 | 0.107512 | 0.017063 | 0.00421 | 0.122106 | -0.25061 | -0.03848 | 0.114804 | 0.098695 | 0.00626 | 1.8E-05 | 0.077396 | 0.080074 | 0.115023 | 0.165551 | 0.086752 | 0.085785 | -0.02901 | 0.066374 | 0.042049 | 0.083718 |
| Pl | -0.14358 | | -0.38262 | -0.01936 | 0.190559 | -0.28929 | -0.2224 | 0.035084 | 0.054968 | -0.00027 | -0.08519 | -0.13163 | -0.04006 | -0.15694 | 0.036396 | 0.107469 | 0.169811 | 0.243381 | 0.428588 | -0.12454 | 0.00978 | -0.04175 | 0.109774 | -0.18365 | 0.053572 | 0.144576 | -0.00906 | 0.157704 | 0.049479 |
| Wa | 0.998965 | | 0.686315 | 0.632087 | 1.425117 | 0.324933 | 0.278242 | 0.108228 | 0.179834 | 0.365042 | 0.089811 | 0.125904 | 0.47412 | 0.137086 | 0.195172 | 0.117326 | 0.253482 | 0.21588 | 0.175326 | 0.058086 | 0.439561 | 0.256596 | 0.248573 | 0.383796 | 0.110566 | 0.216635 | 0.29004 | 0.188142 | 0.155101 |
| Normal | 0.065451 | | -0.37074 | -0.12993 | 0.290718 | -0.16739 | -0.33919 | -0.05933 | -0.17439 | -0.18326 | -0.28186 | -0.49482 | -0.12376 | -0.24031 | -0.11192 | -0.14722 | -0.11002 | 0.025519 | -0.01035 | -0.19633 | -0.11265 | -0.28104 | -0.15571 | -0.25491 | -0.06554 | -0.05842 | -0.23613 | -0.09523 | -0.05117 |

Enriched (red) or de-enriched (blue) expression is color-coded for each gene signature by TNBC subtype.

Ac, acinic cell; Ad, adenoid cystic; AMPK, AMP-activated protein kinase; Ba, basal cell adenoma; CAF, cancer-associated fibroblast; DMPK, dystrophia myotonica-protein kinase; M, mesenchymal; Mu, mucoepidermoid; PDGF, platelet-derived growth factor; Pl, pleomorphic; TMA, tissue microarray;
TNBC, triple-negative breast cancer; Wa, Warthin tumor.

**S3 Table.** Statistical comparison of HER3 mRNA and protein expression levels across breast cancer subtypes in TMA, TCGA and CCLE data sets.

|  | **TMA** | | | | | | | | | | |
| --- | --- | --- | --- | --- | --- | --- | --- | --- | --- | --- | --- |
|  | **EdgeSeq** | | | | | **IHC H-Score** | | | | | |
|  | **HER2+** | **TNBC-BL1** | **TNBC-BL2** | **TNBC-LAR** | **TNBC-M** | **HER2+** | **TNBC-BL1** | **TNBC-BL2** | **TNBC-LAR** | **TNBC-M** |  |
| **ER+/PR+/HER2−** | 2.59.E-03 | 2.82.E-07 | 8.03.E-07 | NS | 3.42.E-03 | NS | 1.78.E-05 | 1.80.E-03 | NS | NS |  |
| **HER2+** |  | 4.31.E-05 | 9.82.E-05 | NS | NS |  | 1.73.E-05 | 5.02.E-04 | 2.86.E-02 | NS |  |
| **TNBC-BL1** |  |  | N.S. | 2.49.E-03 | 7.98.E-03 |  |  | NS | 1.29.E-02 | 1.23.E-03 |  |
| **TNBC-BL2** |  |  |  | 3.89.E-03 | 9.56.E-03 |  |  |  | NS | 3.98.E-02 |  |
| **TNBC-LAR** |  |  |  |  | NS |  |  |  |  | NS |  |
|  | **TCGA** | | | | | | | | | | |
|  | **RNA-seq** | | | | | **RPPA** | | | | | |
|  | **HER2+** | **TNBC-BL1** | **TNBC-BL2** | **TNBC-LAR** | **TNBC-M** | **HER2+** | **TNBC-BL1** | **TNBC-BL2** | **TNBC-LAR** | **TNBC-M** |  |
| **ER+/PR+/HER2−** | NS | 4.08.E-10 | 1.84.E-11 | 4.39.E-05 | NS | NS | 1.13.E-05 | 7.46.E-07 | 1.17.E-03 | NS |  |
| **HER2+** |  | 8.82.E-09 | 1.33.E-10 | 8.60.E-04 | NS |  | 1.46.E-05 | 2.52.E-06 | 2.54.E-03 | NS |  |
| **TNBC-BL1** |  |  | 3.05.E-03 | 5.13.E-03 | 3.79.E-04 |  |  | 1.92.E-02 | NS | 9.65.E-06 |  |
| **TNBC-BL2** |  |  |  | 4.63.E-08 | 5.21.E-07 |  |  |  | 1.03.E-02 | 1.85.E-05 |  |
| **TNBC-LAR** |  |  |  |  | NS |  |  |  |  | 1.23.E-03 |  |
|  | **CCLE** | | | | | | | | | | |
|  | **RNA-seq** | | | | | **RPPA** | | | | | |
|  | **HER2+** | **TNBC-BL1** | **TNBC-BL2** | **TNBC-LAR** | **TNBC-M** | **HER2+** | **TNBC-BL1** | **TNBC-BL2** | **TNBC-LAR** | **TNBC-M** |  |
| **ER+/PR+/HER2−** | NS | 6.46.E-04 | 6.80.E-06 | 4.18.E-02 | 2.20.E-02 | NS | 3.65.E-02 | 1.36.E-05 | 1.32.E-02 | 2.20.E-02 |  |
| **HER2+** |  | 1.55.E-03 | 1.07.E-05 | NS | 1.47.E-02 |  | NS | 6.88.E-05 | 2.73.E-02 | 2.94.E-02 |  |
| **TNBC-BL1** |  |  | N.S. | NS | NS |  |  | NS | NS | NS |  |
| **TNBC-BL2** |  |  |  | NS | NS |  |  |  | NS | NS |  |
| **TNBC-LAR** |  |  |  |  | NS |  |  |  |  | NS |  |

*p* Values (Wilcoxon rank sum test) are shown.
BL, basal-like; CCLE, Cancer Cell Line Encyclopedia; ER, estrogen receptor; HER, human epidermal growth factor receptor; IHC, immunohistochemistry; LAR, luminal androgen receptor; M, mesenchymal; NS, not significant (*p* > 0.05); PR, progesterone receptor; TCGA, The Cancer Genome Atlas;
TMA, tissue microarray; TNBC, triple-negative breast cancer.

**S4 Table.** Statistical comparison of HER3 mRNA and protein expression levels across ovarian cancer subtypes in TMA and CCLE data sets.

|  | **TMA** | | | | | | |
| --- | --- | --- | --- | --- | --- | --- | --- |
|  | **EdgeSeq mRNA** | | |  | **IHC H-Score** | | |
|  | **Mucinous** | **Endometrioid** | **Serous** | **Mucinous** | | **Endometrioid** | **Serous** |
| **Clear cell** | 3.87E-02 | NS | NS | 2.95E-02 | | NS | 8.23E-05 |
| **Mucinous** |  | 9.30E-03 | 8.12E-03 |  | | 3.63E-02 | NS |
| **Endometrioid** |  |  | 3.81E-02 |  | |  | 1.85E-08 |
|  | **CCLE** | | | | | | |
|  | **RNA-seq mRNA** | | | **RPPA** | | | |
|  | **Mucinous** | **Endometrioid** | **Serous** | **Mucinous** | | **Endometrioid** | **Serous** |
| **Clear cell** | NS | NS | NS | NS | | NS | NS |
| **Mucinous** | NS | NS | NS | NS | | NS | NS |
| **Endometrioid** |  | NS | NS |  | | NS | NS |

*p* Values (Wilcoxon rank sum test) are shown.
CCLE, Cancer Cell Line Encyclopedia; HER, human epidermal growth factor receptor; IHC, immunohistochemistry; NS, not significant (*p* > 0.05);
TMA, tissue microarray.

**S1 Figure.** Correlations of EdgeSeq and RNA-seq gene expression data.

**(A)** Correlations of EdgeSeq and RNA-seq gene expression data as an average among 94 cell lines and for *HER2*, *HER3*, and *PD-L1*


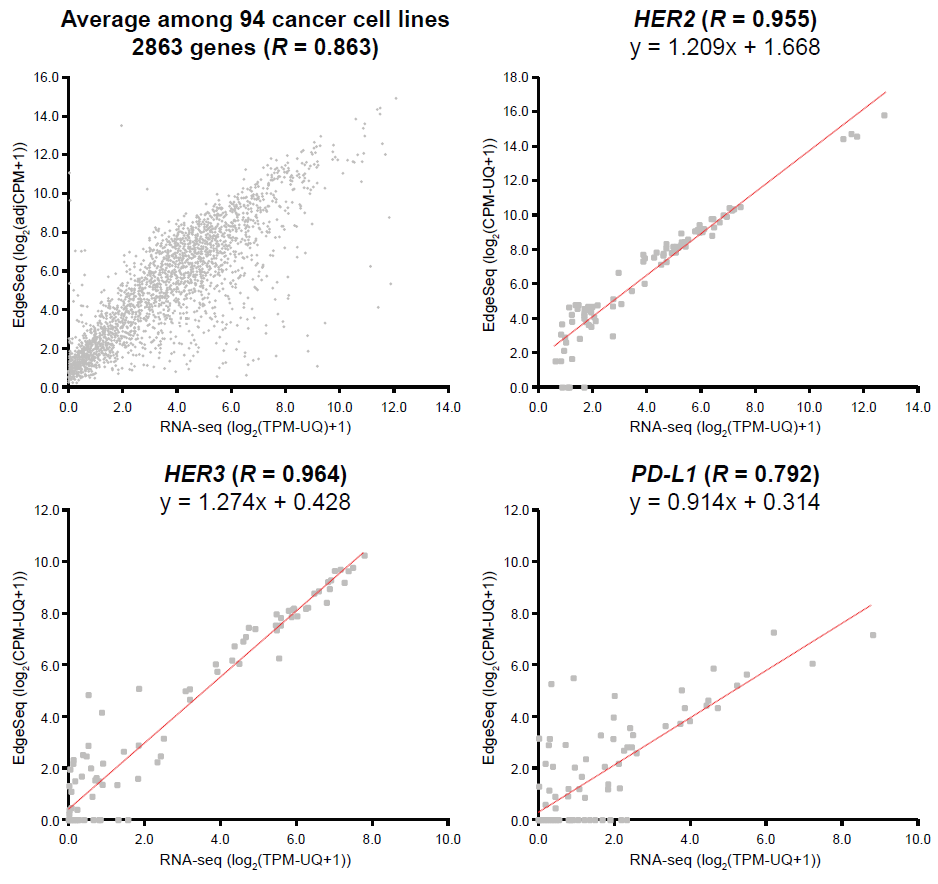


**(B)** Genes with EdgeSeq or RNA-seq biased expression pattern among 94 cancer cell lines, 2863 genes.


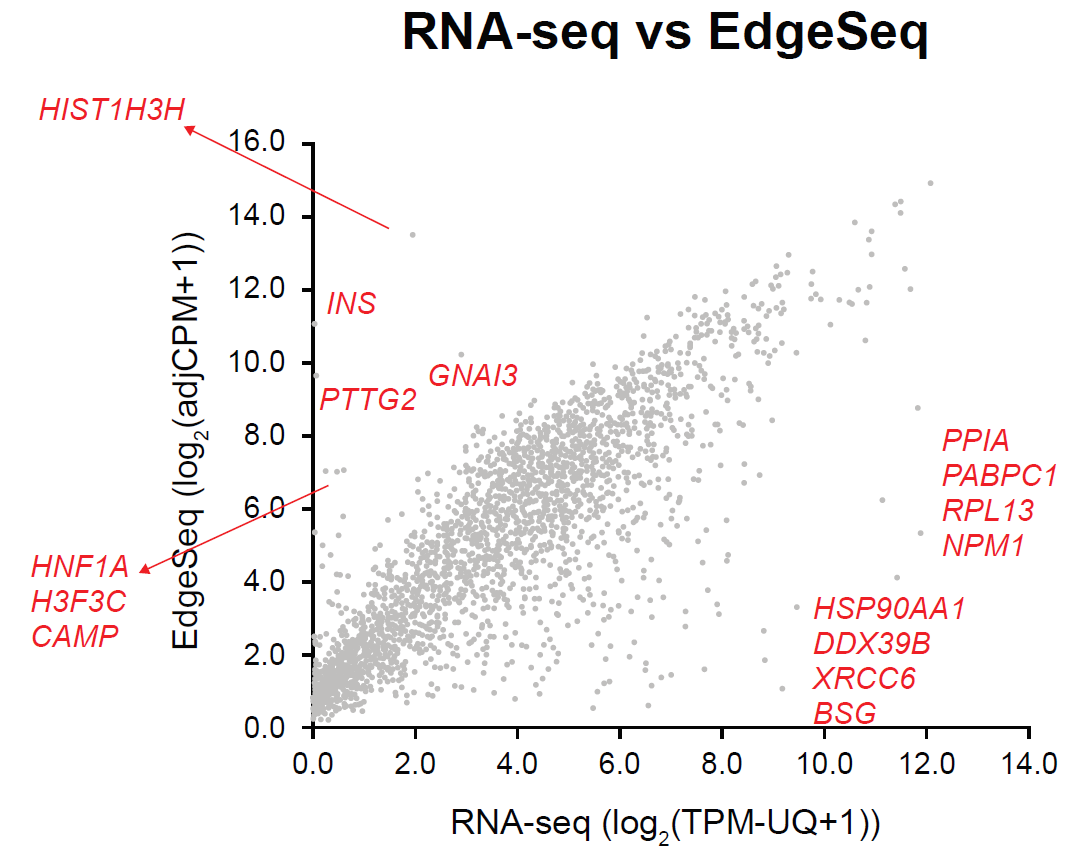


**(C)** Correlations of EdgeSeq and RNA-seq mRNA data in cell lines across the *HER* family, oncogenes and immune markers.


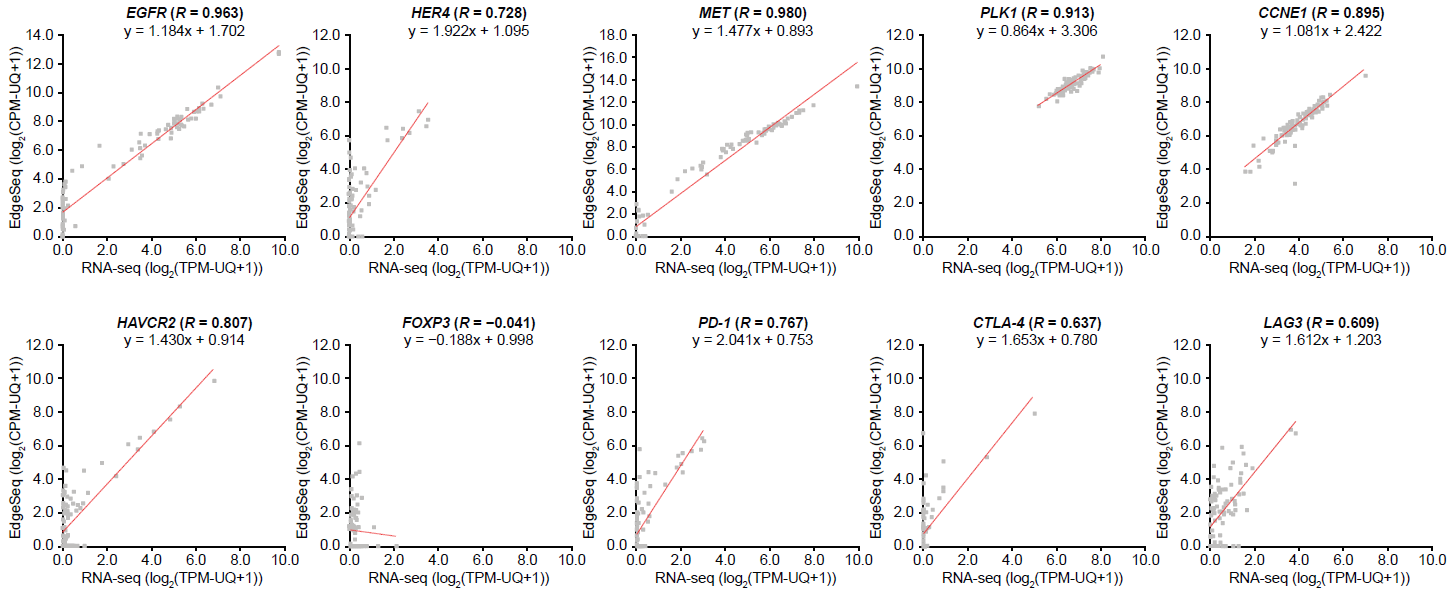


EGFR, epidermal growth factor receptor; HER, human epidermal growth factor receptor; PD-L1, programmed death-ligand 1.

**S2 Figure.** Principal component analysis.


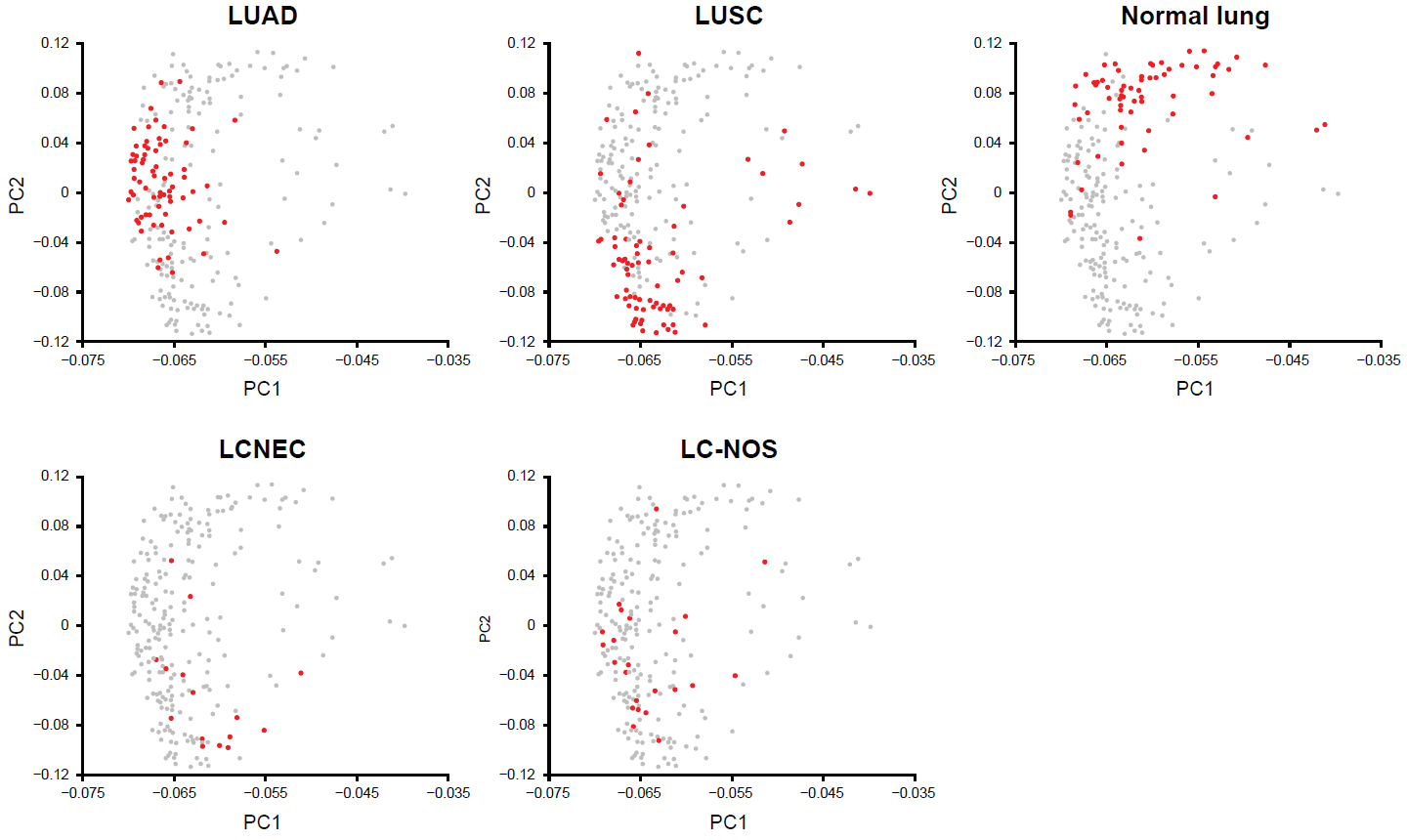
**(A)** Lung cancer.


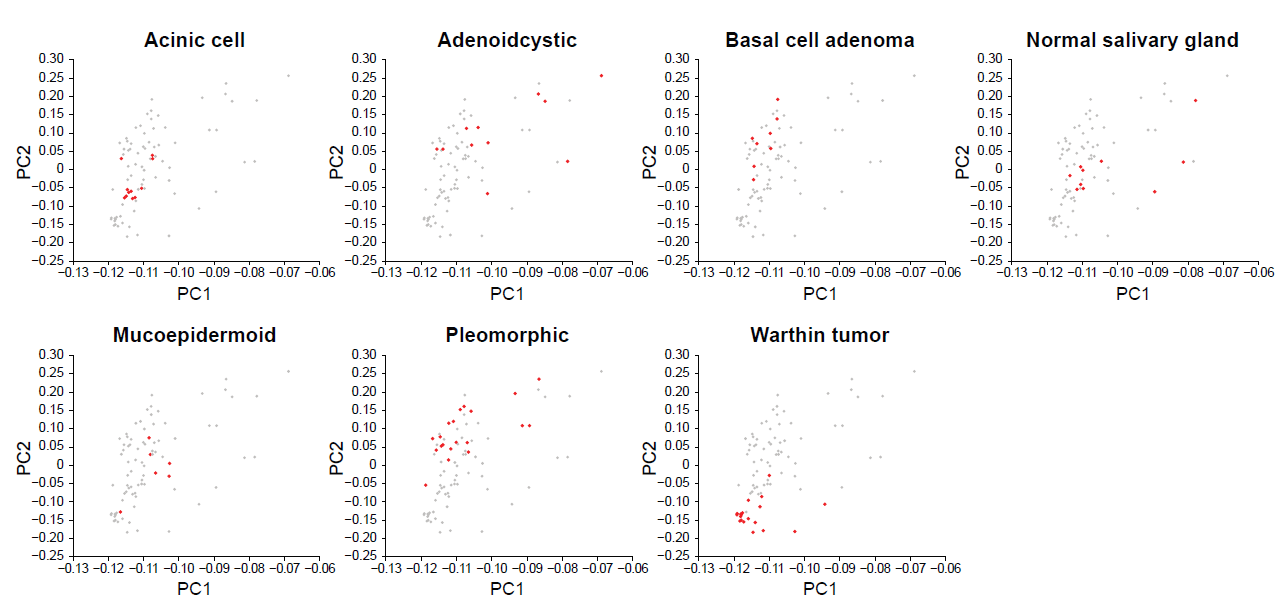
**(B)** Salivary gland cancer.

LC-NOS, large cell lung carcinoma not otherwise specified; LCNEC, large cell neuroendocrine carcinoma;
LUAD, lung adenocarcinoma; LUSC, lung squamous carcinoma.

**S3 Figure.** Expression levels of immune-oncology genes in salivary gland cancer subtypes.


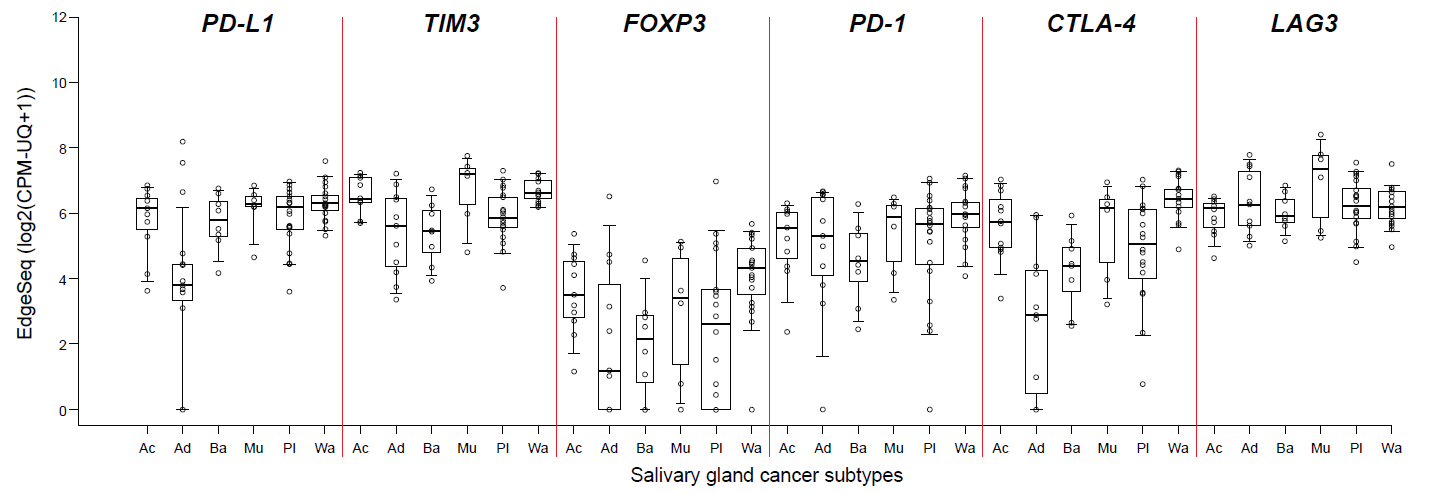


For the box-and-whisker plot, the line in box represents the median and whiskers indicate 5th and 95th percentiles.

Ac, acinic cell; Ad, adenoid cystic; Ba, basal cell adenoma; Mu, mucoepidermoid; PD-1, programmed cell death 1; Pl, pleomorphic; Wa, Warthin tumor.

**S4 Figure.** Correlation analysis of mRNA and protein expression of HER2 and HER3.

**(A)** HER2 mRNA (EdgeSeq) and protein (IHC H-score) correlation in the TMA data set.


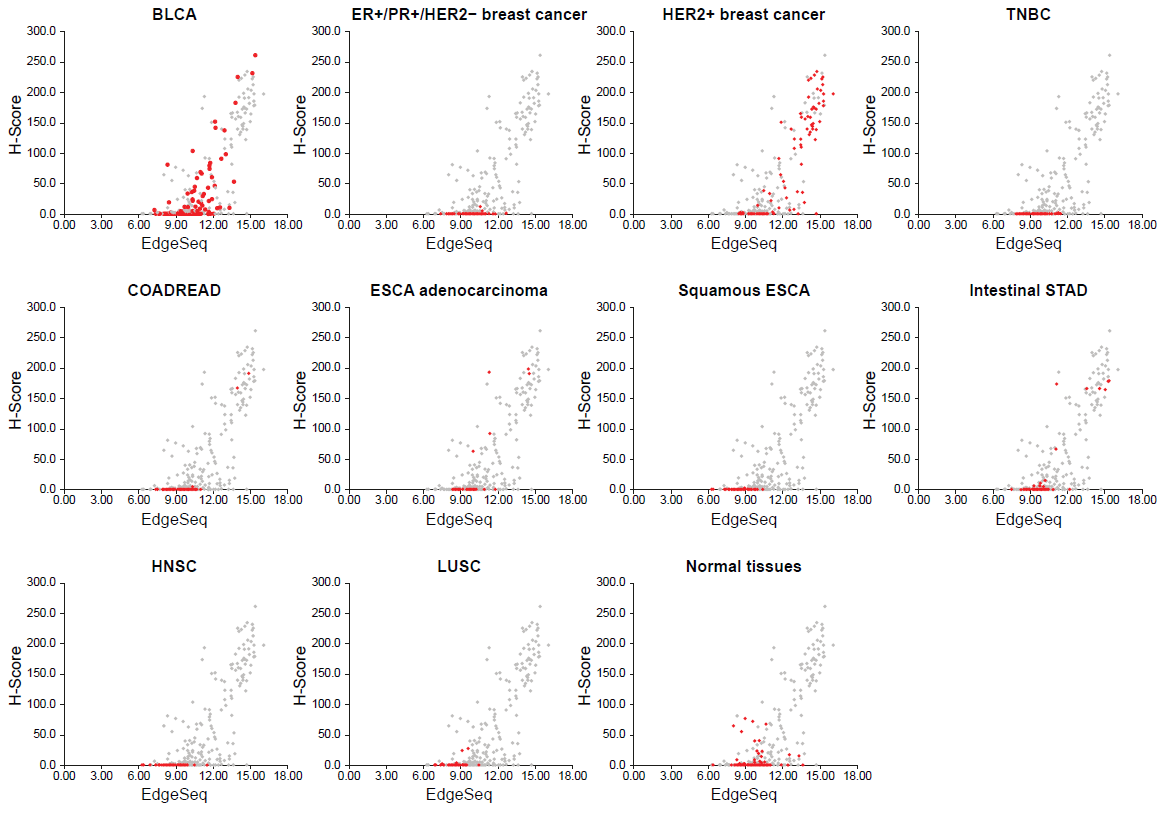


**(B)** HER3 mRNA and protein correlation in LIHC and clear cell ovarian cancer for TMA and CCLE data sets.


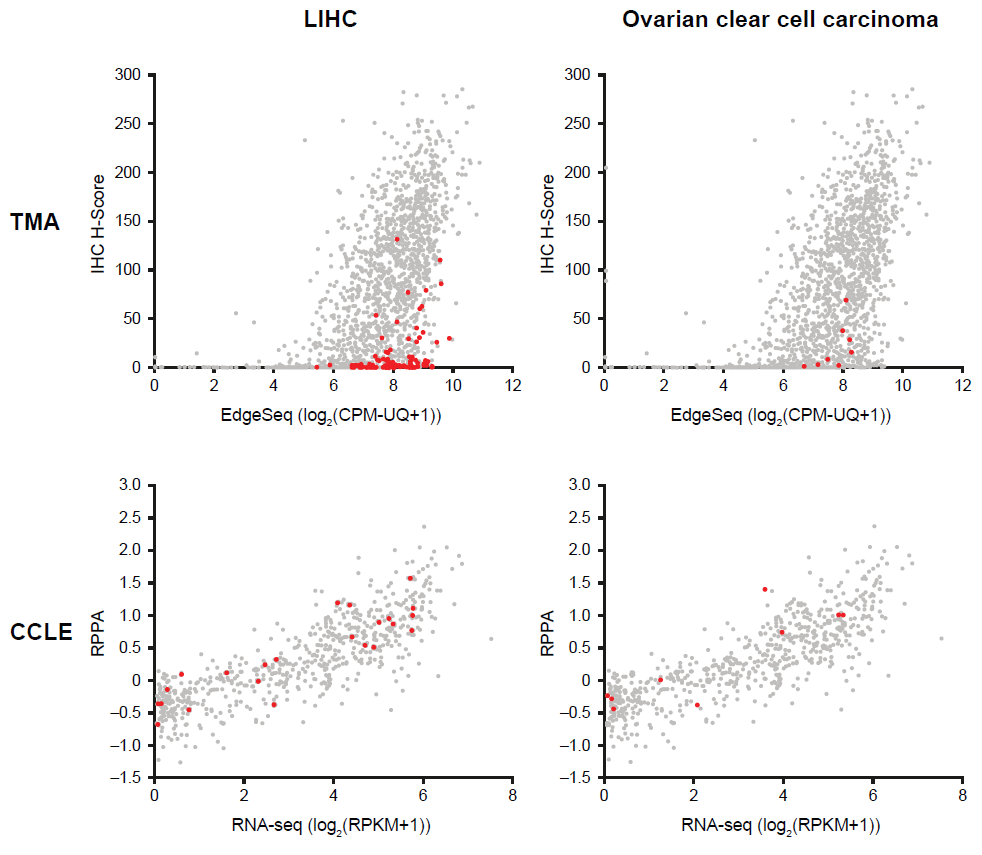


Gray dots indicate all samples, while red dots indicate samples in each cancer type.

BLCA, bladder carcinoma; CCLE, Cancer Cell Line Encyclopedia; COADREAD, colorectal adenocarcinoma;
ER, estrogen receptor; ESCA, esophageal cancer; HER, human epidermal growth factor receptor; HNSC, head-neck squamous cell carcinoma; IHC, immunohistochemistry; LIHC, liver hepatocellular carcinoma; LUSC, lung squamous cell carcinoma; PR, progesterone receptor; STAD, stomach adenocarcinoma; TMA, tissue microarray; TNBC, triple-negative breast cancer.

**S5 Figure.** Coexpression of *HER2* and neighboring genes within the *HER2* expression amplicon.


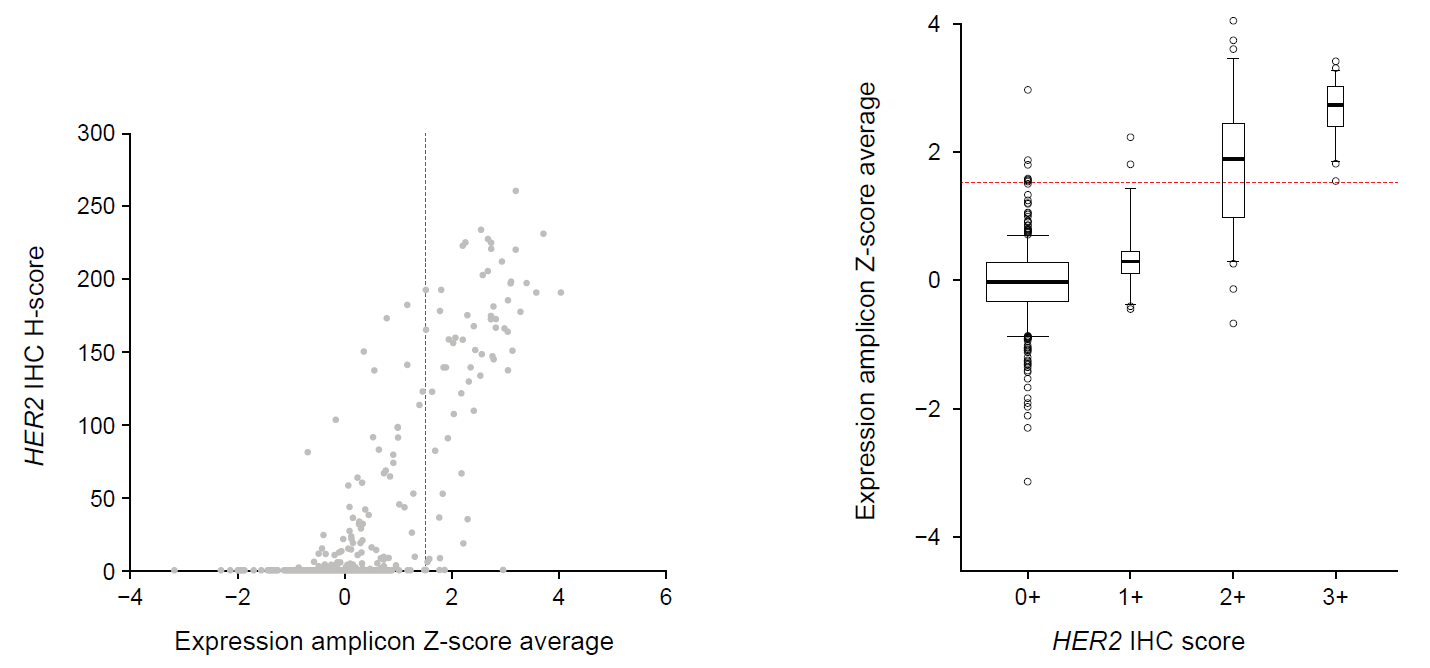


Red dotted line indicates an average Z-score of 1.5, the cutoff selected to define the expression amplicon. For the box-and-whisker plot, the line in box represents the median and whiskers indicate 5th and 95th percentiles.

HER, human epidermal growth factor receptor; IHC, immunohistochemistry.

**S6 Figure.** Correlation analysis of *HER2* and *HER3* mRNA in various tumor types.

**(A)** TMA EdgeSeq.


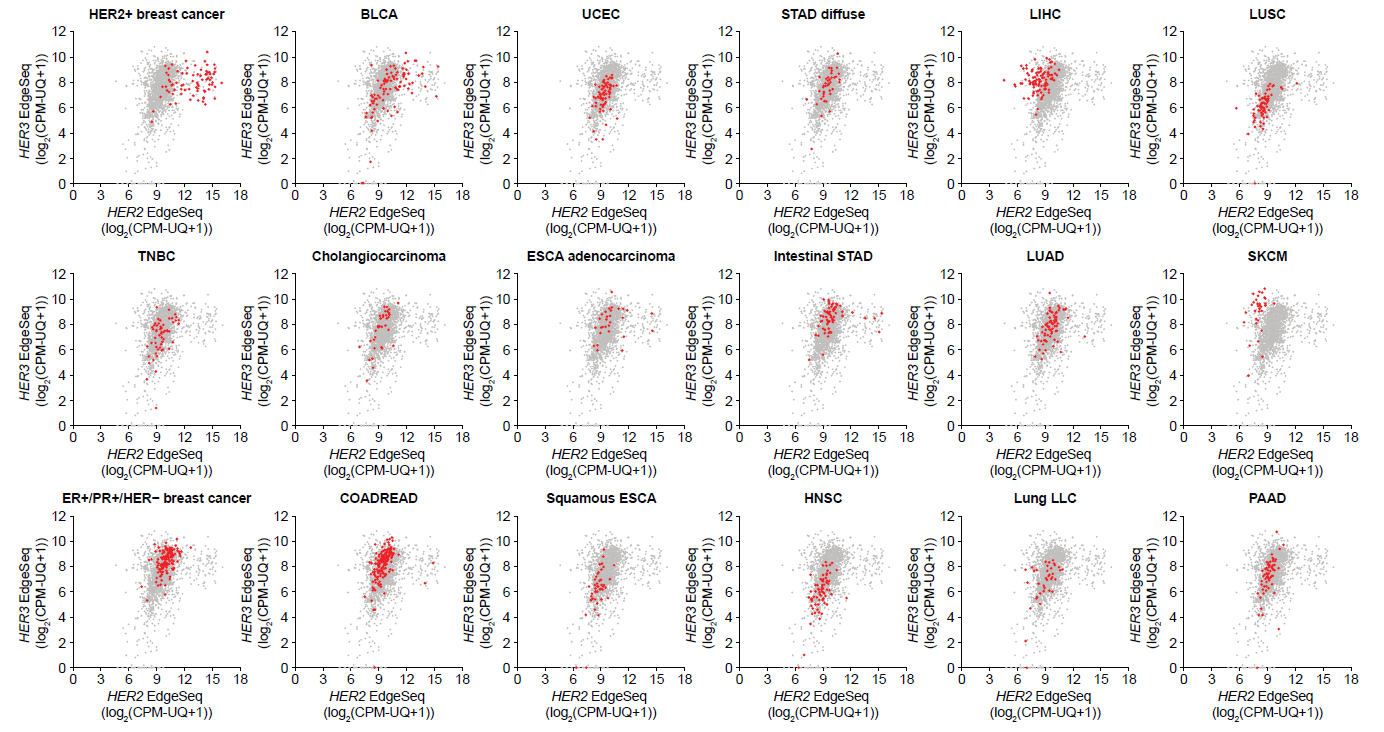


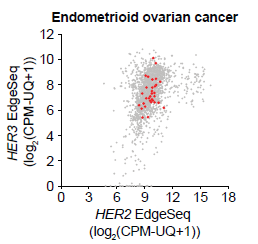

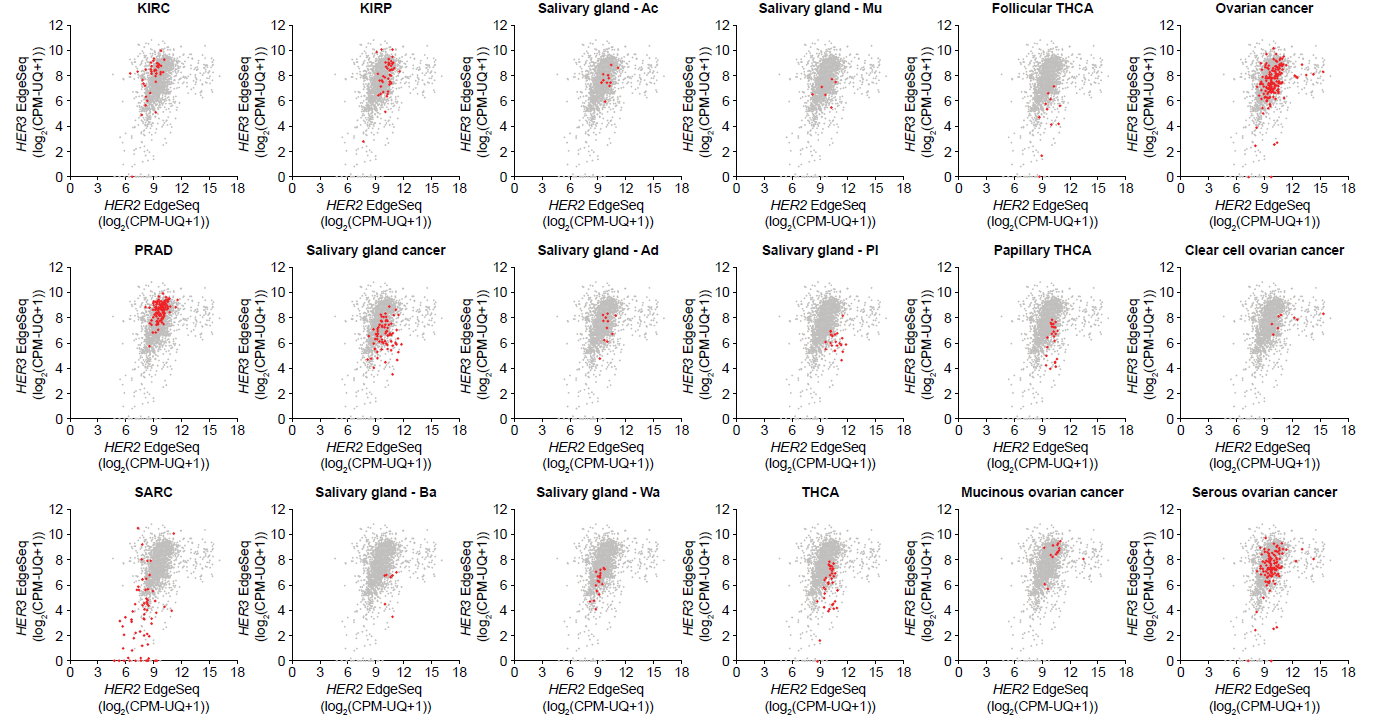


**(B)** TCGA RNA-seq and CCLE RNA-seq.


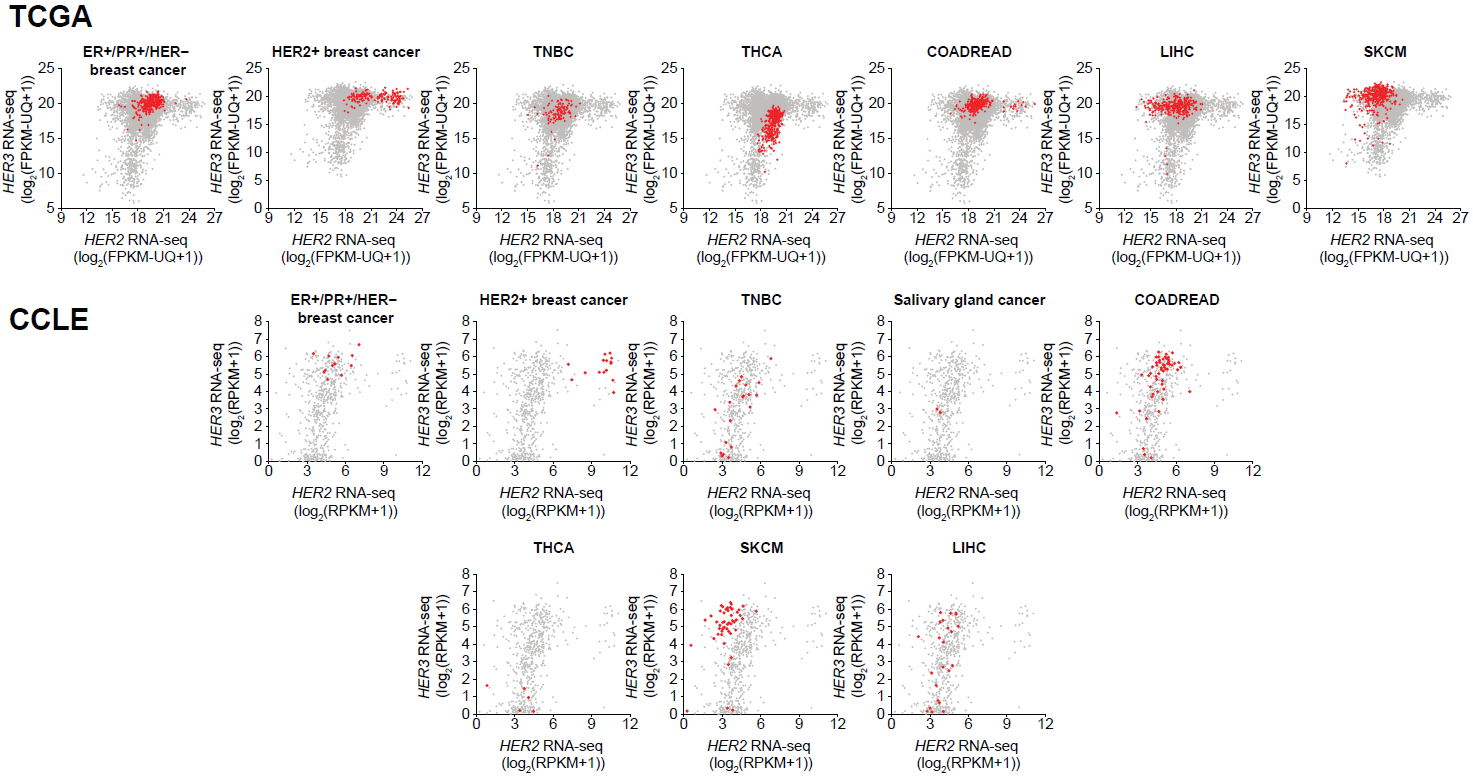


BLCA, bladder carcinoma; CCLE, Cancer Cell Line Encyclopedia; COADREAD, colorectal adenocarcinoma; ER, estrogen receptor; ESCA, esophageal cancer; HER, human epidermal growth factor receptor; HNSC, head-neck squamous cell carcinoma; IHC, immunohistochemistry; KIRC, kidney renal clear cell carcinoma; KIRP, kidney renal papillary cell carcinoma; LIHC, liver hepatocellular carcinoma; LUAD, lung adenocarcinoma; LUSC, lung squamous cell carcinoma; PAAD, pancreatic adenocarcinoma; PR, progesterone receptor; PRAD, prostate adenocarcinoma; STAD, stomach adenocarcinoma; TGCA, The Cancer Genome Atlas; THCA, thyroid cancer; TMA, tissue microarray; TNBC, triple-negative breast cancer; UCEC, uterine corpus endometrial carcinoma.

**Supplemental Methods**

*Lung Cancer Classification*

In general, classification of samples entering this study aligned with the 8th edition of the American Joint Committee on Cancer’s staging of lung cancer, 2018. All core samples included in this study are a representative part of a resected specimen, the diagnosis based on the overall tissue received at the time of primary surgery. Tissue and cellular morphology defined lung adenocarcinoma (LUAD) or lung squamous carcinoma (LUSC), and immunohistochemistry was performed in instances of non–small cell lung cancer. Staining for thyroid transcription factor-1 or p40 defined whether tissue favored LUSC versus LUAD. Epidermal growth factor receptor and anaplastic lymphoma kinase testing were regularly performed in instances of stage IV non–small cell lung cancer, regardless of clinical/pathological features, such as smoking, adenocarcinoma histology or thyroid transcription factor-1 status.

*The Cancer Genome Atlas (TCGA) Cancer Subtype Definition*

Among TCGA breast cancer data, subtypes were defined using clinical information (Phenotype data, version 11-27-2017) to identify human epidermal growth factor receptor 2 (HER2) immunohistochemistry status (positive or negative) and estrogen receptor (ER) and progesterone receptor (PR) immunohistochemistry status (positive or negative). Based on these assessments, the three subtypes were defined as HER2+, ER+/PR+/HER2− and triple negative breast cancer (TNBC; HER2−, ER− and PR−).

Subtypes for TCGA esophageal cancer (ESCA) data were defined based on primary diagnosis. Esophageal cancer adenocarcinoma (ESCA Ad) includes adenocarcinoma not otherwise specified (NOS), mucinous adenocarcinoma and tubular adenocarcinoma. Squamous esophageal cancer includes basaloid squamous cell carcinoma, squamous cell carcinoma, keratinizing NOS and squamous cell carcinoma NOS.

Among TCGA stomach adenocarcinoma (STAD) data, subtypes were defined using clinical phenotype data. Intestinal STAD includes adenocarcinoma-intestinal type, papillary adenocarcinoma NOS and tubular adenocarcinoma. Diffuse STAD includes carcinoma-diffuse type and signet ring cell carcinoma.

For TCGA thyroid cancer (THCA) data, all samples except TCGA-EM-A2CM-01A, TCGA-BJ-A0ZF-01A, TCGA-DJ-A3VJ-01A, TCGA-DJ-A4UQ-01A, TCGA-IM-A41Y-01A and TCGA-IM-A4EB-01A were defined as papillary subtype (THCA_papillary) using clinical phenotype data.

*Subtype Definition by Gene Signature Analysis*

Among the TNBC samples, four subtypes (basal-like 1 [BL1], basal-like 2 [BL2], luminal androgen receptor [LAR] and mesenchymal [M]) were defined by gene signature analysis. Genes within the EdgeSeq panel were selected using TCGA TNBC data (123 samples). Among the samples, 113 had TNBC subtype data, which were obtained from a reference paper (<https://www.ncbi.nlm.nih.gov/pubmed/27310713>). Genes were selected to discriminate each subtype from the others as follows: (1) Z-scores of gene expression levels (log_2_ (FPKM-UQ + 1)) among the all TNBC data were calculated; (2) student *t*-test *p* values were calculated using the Z-score values for all pairs among four subtypes (total six pairs); (3) selected genes for each subtype which show *p* < 0.05 to all of the other subtypes; (4) excluded genes that also show *p* < 0.05 in the other three pairs (i.e., discriminator of the other subtypes); (5) plus or minus direction, which means over- or under-expressed in a given subtype, respectively, was defined for each gene based on averages of Z-scores; and (6) for minus direction genes, the signs of Z-scores were inverted. Averages of the processed Z-scores among the selected genes for each subtype were calculated and defined as subtype scores. The subtypes of each sample were defined by taking a max value among four subtypes for all 123 samples. Using the same gene sets, the same processes were applied to EdgeSeq TMA data (62 samples) to define TNBC subtypes of each sample.

For ovarian cancer, a set of 140 subtype-discriminating genes were selected (Supplementary Data) based on the average gene expression level in an indicated subtype being higher (log2 > 2.0) or lower (log2 < –2.0) than samples of other subtypes and t-test *p* value is < 0.005. Z score was calculated for each expressed gene, among all samples. Averages of Z score among each subtype discriminating gene was calculated for each subtype (clear cell, endometrioid, mucinous and serous). For genes whose expression levels are negative and lower than other subtypes, the minus sign of the Z score of subtype discriminating genes was converted to plus and the positive values were used for calculating the average (Supplementary Data). Among the four subtypes in each sample, one subtype with the highest Z score average was defined as the mRNA subtype.

For lung cancer, a set of 77 subtype-discriminating genes were selected (Supplementary Data). Lung adenocarcinoma and lung squamous carcinoma discriminating genes were higher (log_2_ > 2.0) in average gene expression than in the other subtypes (*p* < 0.005). Large cell neuroendocrine carcinoma genes were higher (log_2_ > 2.0) or lower (log_2_ < –2.0) in average gene expression than samples of LUAD and LUSC subtypes, respectively (*p* < 0.05). In each sample, one subtype was defined as the mRNA subtype in the same manner as described as above using Z scores.

For thyroid cancer, a set of 85 subtype-discriminating genes were selected (Supplementary Data). Papillary subtype genes were defined by two parameters: higher (log_2_ > 1.0) or lower (log_2_ < –1.0) average gene expression than samples from both the follicular subtype and normal thyroid tissue (*p* < 0.05), and removal of genes with higher or lower expression in the follicular subset, compared with normal thyroid tissue (*p* < 0.05). The same approach was taken to identify follicular subtype genes, but comparisons occurred with the papillary subtype and normal tissue. In each sample, one subtype was defined as the mRNA subtype in the same manner as above using Z scores.

A set of 69 subtype-discriminating genes were selected for gastric cancer (Supplementary Data). Genes to identify the intestinal and diffuse gastric cancer subtypes were selected in a comparable manner as described above for thyroid cancer (i.e., higher [log2 > 2.0] or lower [log2 < –2.0]) average gene expression during cancer subtype/normal tissue comparisons. In each sample, one subtype was defined as the mRNA subtype in the same manner as described above for lung cancer, using Z scores.

For salivary tumors, a set of 523 subtype and normal salivary tissue-discriminating genes were selected (Supplementary Data). Each tissue discriminating gene was higher (log2 > 2.0) or lower (log2 < –2.0) in average gene expression compared with the other samples (*p* < 0.05). In each sample, one subtype was defined as the mRNA subtype in the same manner as above, using Z scores.

*Edgeseq Gene Expression Analysis and Data Processing*

In HTG EdgeSeq technology, the next generation sequencing library was constructed using automated processing machines directly from TMA slices. Internal controls for Edgeseq analysis consisted of four positive and 16 negative controls (5 ‘NEG_CTRL_ANT’, 10 ‘ER-‘, and ‘metrn’ probes) and control RNAs (universal RNA or multitissue control), which were placed in each processing plate.

Relative standard deviation was defined as s/, where s and are the standard deviation and average of log_2_ (count + 1), respectively. In several of the samples remaining after quality filtering (2744 of 3033 sequenced samples), high background of negative control read counts was observed, potentially due to insufficient digestion of nonhybridized probes by S1 nuclease. Subtraction of background values from all gene probes was conducted as follows: (1) calculated averages of 16 negative control read counts; (2) calculated adjusted total gene count (adjusted total gene count = total read count – counts of positive and negative controls – [average of negative controls × 2867]); (3) calculated adjusted count-per-million (adjCPM) expression values (each gene probe’s count - average of negative controls) / adjusted total gene count × 1,000,000; (4) minus values of adjCPM were transformed to zero.

Upper-quartile (UQ) normalization was applied to reduce tissue-specific bias of the expression data and was calculated as follows: (1) UQ value of adjCPM from expressing genes (expression value > 0) in each sample; (2) median of the UQ values among 2744 samples that passed the sequencing quality filter; and (3) adjCPM-UQ (adCPM-UQ = each gene’s adjCPM / each sample’s UQ × median of UQ).

Samples with low correlations (*R* < 0.4) were considered low quality and a sample quality filter was applied by empirically setting an exclusion parameter based on average of expression values (log2 [adjCPM-UQ + 1]) of nine house-keeping genes (*DDX5, EEF1G, GAPDH, NCL, RPL38, RPS7, SLC25A3, SOD1, YWHAZ*) being less than 11.0.

*Comparison With TCGA RNA-seq Data and In-House Cell Line RNA-seq Data*

RNA-seq data (fragments per kilobase of transcript per million mapped reads upper quartile) from TCGA were downloaded from UCSC-Xena (<https://xenabrowser.net/>). The Human Genome build 38 (hg38) data set was used by TCGA and in-house RNA-seq. The following probes were not used in the comparison of EdgeSeq with TCGA or with in-house RNA-seq data: IFNA-Family (no symbol in hg38); WHSC1L1 (duplicated probe, NSD3); TREX1, CDH3, and HSPA6 (probes are not matched to hg38 RefSeq region).

*RNA-seq Analysis*

After mRNA isolation, complementary DNAs (cDNAs) were generated. After fragmentation of mRNAs by incubation at 94°C for 15 minutes, first-stranded cDNAs were synthesized at 42°C for 15 minutes. Second-stranded DNAs were generated at 16°C for 60 minutes, followed by end-repair reaction at 20°C for 30 minutes. Adaptor-DNA oligomers were ligated at 20°C for 15 minutes, and cDNAs were purified and size selected with Agencourt AMPure XP beads (Beckman Coulter, Brea, CA, USA). cDNA was amplified by polymerase chain reaction (98°C for 30 seconds, 12 cycles of 98°C for 10 seconds, 65°C for 30 seconds, 72°C for 30 seconds followed by 72°C for 5 minutes) using KAPA HiFi Hot Start Ready Mix (Roche, Basel, Switzerland) and was purified with Agencourt AMPure XP beads (Beckman Coulter). The cDNA libraries were pooled and incubated with 0.1N NaOH for 5 minutes at room temperature, followed by dilutions to 1.2-2.4 pM with hybridization buffer (illumina; San Diego, CA, USA), according to manufacturer’s protocol. Paired-end-read sequencings (150 bp) were performed using NextSeq500 or 550 (illumina). Base call files obtained by NextSeq Control Software and RTA (Real Time Analysis) were transformed to fastq files using bcl2fastq v2.20.0.422. Reads in the fastq files were aligned with STAR_2.5.3a to transcript references based on Gencode human release 23 (GRCh38.p3) and read counts in each gene were estimated by RSEM_1.3.0. Gene expression levels were presented as transcripts per kilobase million (TPM) values. TPM data were UQ normalized, and log2 (TPM-UQ + 1) data were compared with EdgeSeq data.

*Immunohistochemistry*

HER2 immunohistochemical staining was performed on the formalin-fixed, paraffin embedded slides using PATHWAY anti-HER2/neu (4B5; rabbit monoclonal; predilution) antibody and an ultraView Universal DAB kit on an automatic immunostainer BenchMark XT, following the manufacturer’s guidelines. All used reagents and instruments were from Ventana Medical Systems (Tucson, AZ, USA).

HER3 immunohistochemical staining was performed on an automatic immunostainer BenchMark XT. After deparaffinization and antigen retrieval with cell conditioning I for 56 minutes at 100°C, the formalin-fixed, paraffin embedded slides were incubated with anti-HER3 antibody (0.5 µg/mL, SPM738;abcam, Waltham, MA, USA) for 16 minutes at 36°C and visualized with an OptiView Universal DAB kit and counterstained with hematoxylin II. All used reagents (other than the primary antibody) and instruments were from Ventana Medical Systems.
